# Supplementary material for: Gut Microbiome Modulation and Health Benefits of a Novel Fucoidan Extract from Saccharina latissima: A Double-Blind, Placebo-Controlled Trial
Source: Microorganisms. 2025 Jun 30;13(7):1545. doi: 10.3390/microorganisms13071545 (PMC12298129; doi:10.3390/microorganisms13071545)
Supplement: Supplementary file 1 [file microorganisms-13-01545-s001.zip › microorganisms-3667635-supplementary.pdf]

SUPPLEMENTARY MATERIAL S1

Preclinical Studies

Experimental details are similar to those described in Laura E. McCoubrey *et al.* 2024 [138] with the following changes and additions.

1. Materials and methods

Note that medium composition of ProDigest's proprietary basal medium is not disclosed and is referred to as PD01. Also, mucin-coated beads were not used in this study.

In vitro fermentation

These experiments were conducted at ProDigest (Gent, Belgium). The human colon model consisted of glass vessels inoculated with 70 mL sterile basal medium, 3.5 g/L Inulin (Sensus), 0.5 g/L, 1.5 g/L or 3.5 g/L seaweed extract, and 10% v/v faecal suspension. Here, the fiber-depleted basal medium contained a proprietary mix of nutrients (ProDigest's nutritional medium PD01) and included a phosphate-buffer to maintain pH in the simulation at physiologically relevant levels.

Analysis of selected post-biotic metabolites

Samples were subjected to the MetaKey® metabolomics platform, using ultra-high performance liquid-chromatography coupled to high-resolution mass spectrometry (UHPLC-HRMS), for relative quantification of 20 polar metabolites linked to cardiac health. Additionally, screening was conducted for two phlorotannins, here identified as Bifuhalol A (identified in A. nodosum) and Bifuhalol B (identified in S. latissima). For these metabolites, no authentic references were included in the study. In this respect, tentative identification was mainly based on the accurate m/z-value, whereby information on the metabolites' mass spectra as defined in Sardari et al. (2020) [139] was consulted to support the identification process.

Phlorotannin	Species	Formula	Theoretical m/z
Bifuhalol A	*Ascophyllum nodosum*	C18H14O10	390.30
Bifuhalol B	*Saccharina latissima*	C12H10O7	266.20

Analytical standards and reagents

Stock solutions of analytical standards and internal standards were prepared at 1 mg mL-1, mostly using ultrapure water and along with derived working solutions were stored at -20 °C in amber glass vials. Solvents used for analysis were of LC-MS grade and from VWR International (Belgium) or Fisher Scientific (USA). Ultrapure water was derived through a purified water system (VWR International, Belgium, 0.055 µS cm-1).

Sample extraction

For analysis, samples were subjected to a liquid-based extraction protocol that was based on the work of Vanden Bussche et al. (2015) [140].

Samples were vortexed for 1 minute at room temperature after which they were centrifuged for 5 min (13,300 x g, room temperature). The resulting supernatant was passed through a polyvinylidene fluoride filter (13 mm diameter, 0.22 µm) (Merck, Ireland) into a 2-mL Eppendorf tube. From this tube, 300 µL fluid was transferred into an LC vial and 600 µL ultrapure water (containing the internal standards) was added. A 5-µL aliquot of each sample was injected into the UHPLC-HRMS system for polar metabolomics analysis. Instrumental analysis Polar metabolomics analysis was performed according to Vanden Bussche et al. (2015) [140] and De Paepe et al. (2018) [141].

Chromatographic separation was achieved on a Vanquish quaternary pumping system (Thermo Fisher Scientific, USA), equipped with an Acquity HSS T3 C18 column (1.8 µm, 150 x 2.1 mm) (Waters Corporation, UK). A binary solvent system consisting of ultrapure water and acetonitrile, both acidified with 0.1% formic acid, was used at a constant flow rate and by applying a gradient profile. Detection was performed on a Q-Exactive™ standalone bench top quadrupole-Orbitrap high resolution mass spectrometer (Thermo Fisher Scientific, USA), which was preceded by heated electrospray ionization (HESI-II source) in polarity switching mode. The instrument was operated at a resolution of 140,000 full width at half maximum and in full-scan mode, meaning that all ions detected were registered and no fragmentation was applied. For the measurement of p-Cresol, a fluorescence detector was coupled (in series) to the mass spectrometer. Measures for quality assurance Prior to analysis, the MS system was calibrated according to the manufacturer's guidelines (Thermo Fisher Scientific) to warrant accurate mass measurements (< 5 ppm mass deviation) in both positive and negative ionization mode. In addition, the chromatographic and mass spectrometry performance were evaluated by injecting a standard mixture, which contained the target metabolites. The biological samples (n = 80) were analyzed in a randomized order within each donor whereby also randomization between donors was applied. Samples per donor were, however, kept together.

Quality control samples (QC) were used to condition the LC-MS instrument as well as perform continuous monitoring of the instrument performance by repeated analysis of the QC-sample. With respect to the latter, so-called internal QC-samples (iQC) were generated based on the pooled material of 10 different samples. Hereby, two types were considered. A first type (iQC1) was enriched with all targeted metabolites (see below, "Panel of targeted metabolites") prior to extraction whereas no enrichment step was considered for the second type (iQC2). Identification and relative quantification of metabolites A metabolite was positively identified in the LC-MS raw data taking into account the i) accurate m/z value of the molecular ion (allowed mass deviation of 5 ppm), ii) 13C isotope pattern, and iii) relative retention time (taking into account the retention time of the nearest eluting internal standard) (allowed time deviation of 2.5%).

Identification and quantification of metabolites

Xcalibur software (Thermo Fisher Scientific, USA) was used. Following peak integration, the area ratio was determined for each metabolite by calculating the ratio between the area of the metabolite and that of the most appropriate internal standard. For the selection of such an internal standard, the similarity in retention time between the targeted metabolite and the internal standard as well as the coefficient of variance (CV%) (as calculated for the iQC samples) were taken into consideration. Subsequently, also the iQC-samples were used for normalization, i.e. to correct for potential instrumental drift during analysis. The final quantitative measure was an iQC-normalized area ratio. In general, a metabolite was considered below the LOQ if the observed peak area was below 150,000. However, information

on the general noise level and stability of retention time were additional features to assess the reliability of detection and quantification. Due to the different method of detection (fluorescence detector), normalization based on an internal standard or on the iQC-samples was not possible for the metabolite p-cresol.

Results

Gas production

Besides pH, gas production is a measure of overall microbial activity, and thus of speed of fermentation. Results on gas production are shown in Figure S1. At concentrations of 0.5 and 1.5 g/L, seaweed extract did not alter gas production upon fermentation as compared to the negative control. However, at the highest concentration of 3.5 g/L, stimulation of gas production was statistically significant on each timepoint. Although the effect was significant and thus consistent across the donors, the increase in gas pressure associated with 3.5 g/L seaweed extract was limited to +3.3 kPa (+13% as compared to negative control by the end of the incubation (p=0.019)), making the effect biologically irrelevant.

In contrast, the fiber control (3.5 g/L chicory inulin) exhibited the highest gas production rate, generating double the amount of gas as compared to the negative control within 48 hours (+25.5 kPa (+98%) as compared to negative control; p=0.0002). Gas production mostly took place between 6–24h. Note that *in vivo* trials have shown that consuming a high dose of chicory inulin (5 g/day, equivalent to approximately 8.33 g/L in our *in vitro* system) is associated with bloating [142].

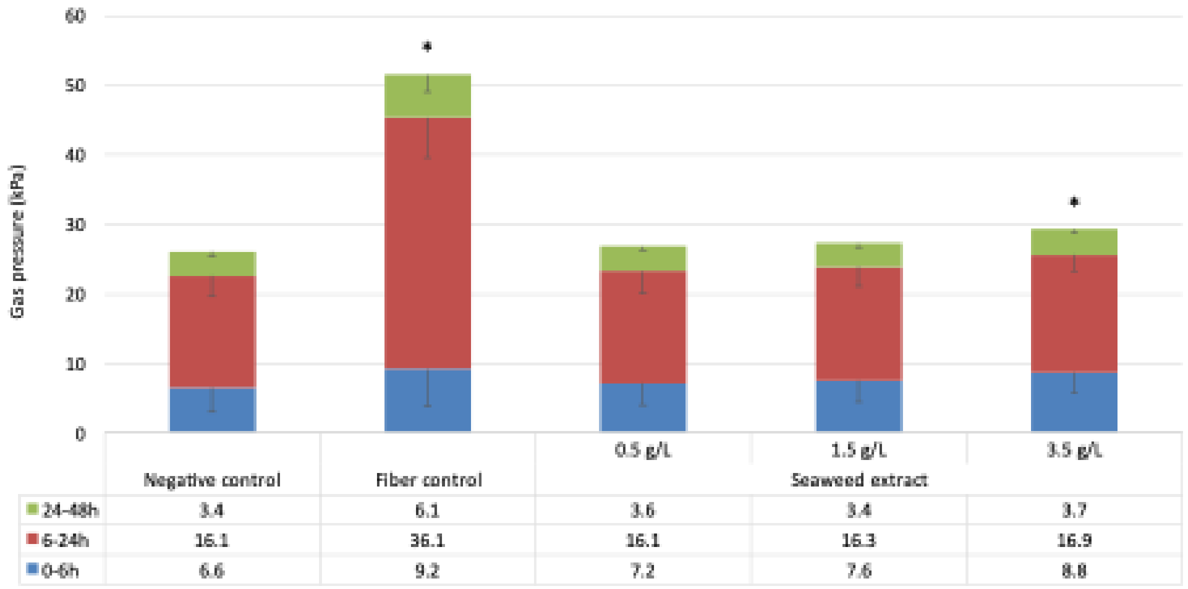

Figure S1: Average gas production across donors (kPa) during different timeframes of the 48h incubation, with error bars as a measure of interindividual variation (standard deviation on average across donors). The seaweed extract was tested in three concentrations (0.5, 1.5 and 3.5 g/L); a fiber control (3.5 g/L inulin) and a negative control (blank) were included as reference conditions. Significant differences in total production (0-48h) between treatment and negative control are indicated with an asterisk (*) (p<0.05).

Changes in microbial metabolites

For each metabolite, two types of figures are displayed. The first one being a bar plot, showing metabolite production in the various conditions during incubation; the second one being a volcano plot, showing the magnitude of change versus respective reference conditions (fold-change, x-axis) in function of statistical significance (p-value, y-axis). The p-value was obtained from paired T-tests between treatment and reference condition, using the individual donors as 'replicates'. The cut-off for statistical significance was set at $p < 0.05$.

Short-chain fatty acids

SCFA production results from carbohydrate metabolism in the colon and is related with various health effects. The most abundantly produced SCFAs include acetate, propionate and butyrate. Whereas acetate can be used as an energy source for the host and as a potential substrate for lipid synthesis in the body, propionate reduces cholesterol and fatty acid synthesis in the liver (beneficial effect on metabolic homeostasis). Butyrate on the other hand, is a major energy source for colonocytes and induces differentiation in these cells (related to cancer prevention). Positive effects of the investigated substrates on SCFA production therefore include increases of acetate, propionate and/or butyrate.

Acetate can be produced by many different gut microbes (including amongst others *Bifidobacterium* spp., *Bacteroides* spp. and *Lactobacillus* spp.) and is a primary metabolite generated from substrate fermentation. Results are shown in Figure S2 (bar plot) and Figure S3 (volcano plot). The seaweed extract stimulated acetate production and a dose-response was observed, with higher concentrations resulting in more acetate. Acetogenic effects were noticed as early as 6h, and significance was reached for the 3.5 g/L concentration (+1.0 mM (+14%) as compared to negative control; p=0.023). By the end of the incubation (0-48h), the lowest seaweed concentration (0.5 g/L) had generated slightly less acetate than the negative control; the 1.5 and 3.5 g/L concentrations generated more acetate than blank in a dose-response manner (+3% and +8%, respectively). However, significance was not reached. The fiber control strongly and significantly stimulated acetate production at each timepoint during incubation, finally resulting in a +13 mM (+72%) increase as compared to negative control (p<0.00001).

Propionate can be produced by different gut microbes, with the most abundant propionate producers being *Bacteroides* spp., *Akkermansia muciniphila* and *Veillonellaceae*. Results are shown in Figure S2 (bar plot) and Figure S4 (volcano plots). Seaweed extract stimulated propionate production, and a dose-response was observed. Initially (0-6h), the highest concentration (3.5 g/L) not only resulted in significantly more propionate than negative control, but also as compared to the fiber control (significance not reached). At 24h, propionate levels had further increased with the seaweed extract, and a dose-response was observed with significance reached for 1.5 g/L and 3.5 g/L. Still, inulin's propionogenic effect was stronger. At the end of incubation (48h), 1.5 g/L and 3.5 g/L seaweed extract had generated significantly more propionate than negative control (+1.1 mM (+19%) and +1.7 mM (+29%) respectively; p=0.030, p=0.006). Inulin, however, resulted in the highest final propionate yield (+3.4 mM (+58%) as compared to negative control; p=0.046), but significance was not reached due toa higher degree of interindividual variation.

Butyrate is mostly produced by members of the *Lachnospiraceae* and *Ruminococcaceae* families. In a process called cross-feeding, these microbes convert acetate and/or lactate

(along with other substrates) to the health-related butyrate. Results on butyrate production are shown in Figure S2 (bar plot) and Figure S5 (volcano plot). Overall, butyrate production was low between 0-6h. Indeed, butyrate is a secondary metabolite produced through cross-feeding of acetate and lactate, and therefore typically produced during late stages of the incubation. The seaweed extract stimulated butyrate production in most donors, and in most cases a dose-response was observed. Significant effects were not observed until 48h, when 1.5 g/L and 3.5 g/L seaweed extract generated significantly more butyrate than negative control (+0.4 mM (+12%) and +0.6 mM (+19%), respectively). The strongest butyrogenic effect was, however, attributed to inulin, but significance was not reached due to higher interindividual variation (+2.0 mM (+58%)). Although on average effect size was bigger for inulin, some donors were not affected by treatment. This is in contrast with seaweed, where a more consistent effect across the donors was noticed, yet with smaller effect size.

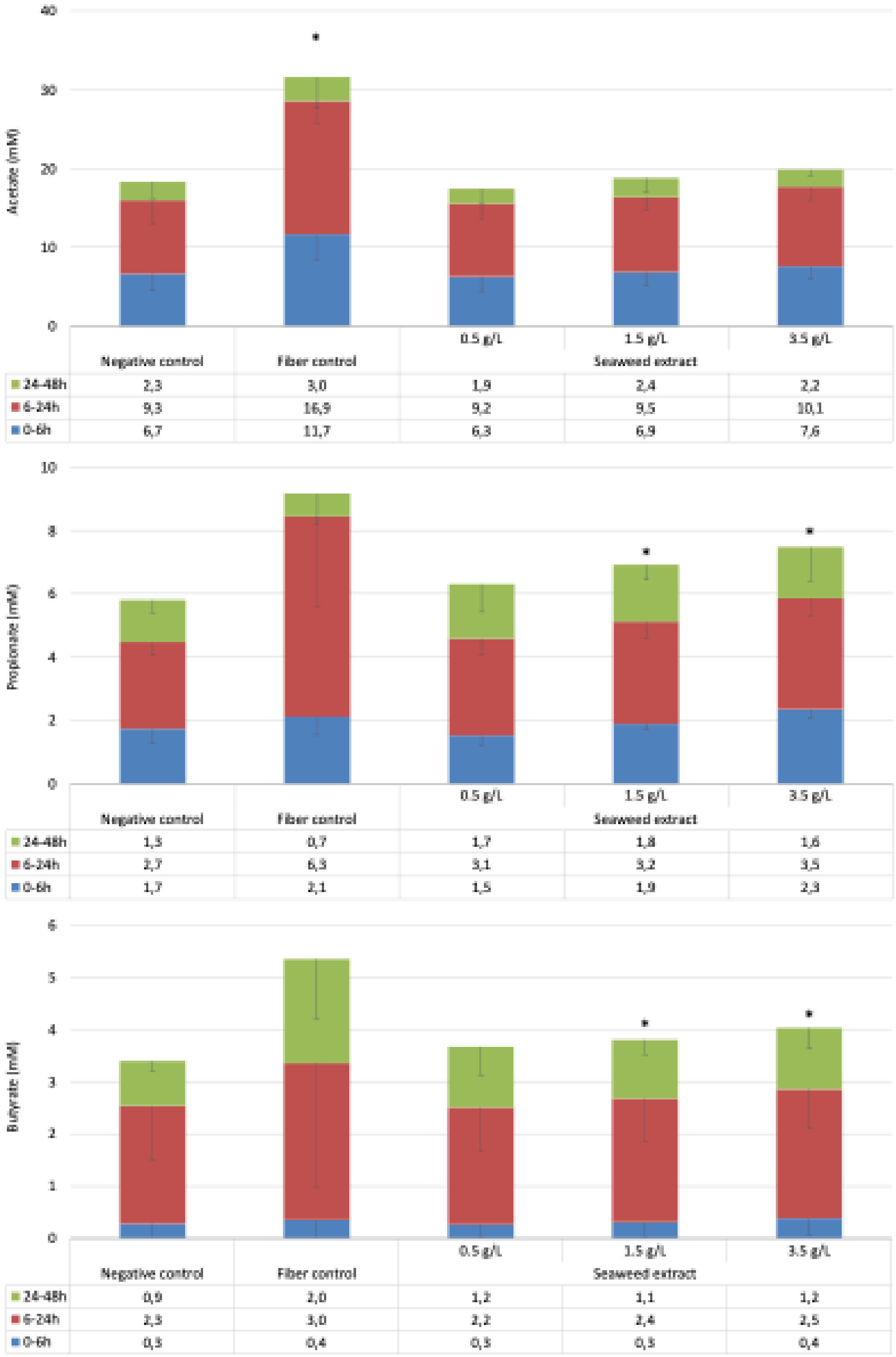

Figure S2: Average acetate (top), propionate (middle) and butyrate (bottom) production across donors (mM) during different timeframes of the 48h incubations, with error bars as a measure of interindividual variation (standard deviation on average across donors). The seaweed extract was tested in three concentrations (0.5, 1.5 and 3.5 g/L); a fiber control

(3.5 g/L inulin) and a negative control (blank) were included as reference conditions. Significant differences between treatment and negative control for across donor comparisons on total production (0-48h) were indicated with an asterisk (*) ($p<0.05$).

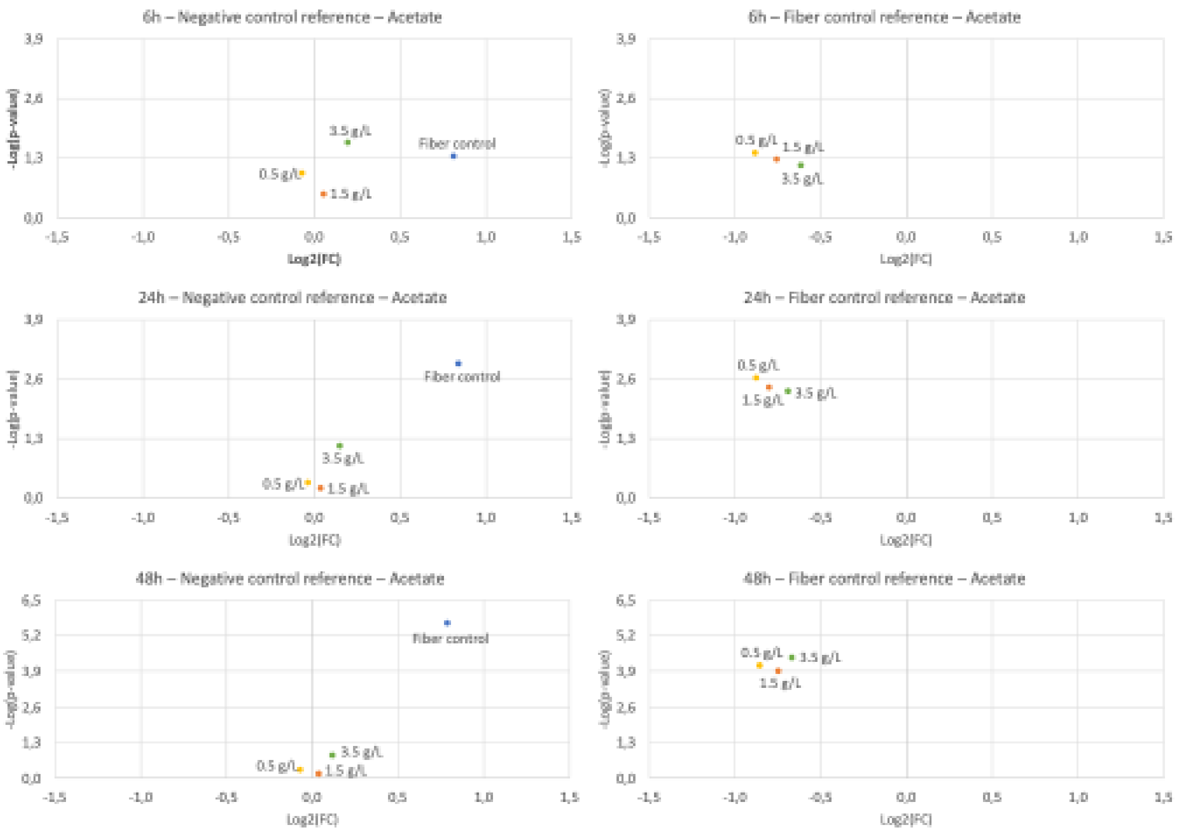

Figure S3: Volcano plot showing differences in acetate production between treatments (0.5, 1.5, 3.5 g/L seaweed extract and fiber control (3.5 g/L chicory inulin)) and negative control (left) and between treatments and the fiber control (right) after 6h, 24h and 48h of incubation across the five healthy donors. Statistical significance (-log(p-value)) is plotted in function of fold change (log2(FC)), classifying treatments into one of four categories: non-significantly lower acetate production than respective reference (bottom left), significantly lower acetate production than respective reference (top left), non-significantly higher acetate production than respective reference (bottom right) and significantly higher acetate production than respective reference (top right). The 1.3 value on the y-axis marks the level above which an effect is statistically significant compared to respective reference condition (corresponding with $p<0.05$). The zero value on the x-axis marks the separation between elevated acetate production versus respective reference condition (right) or lower acetate production than respective reference condition (left).

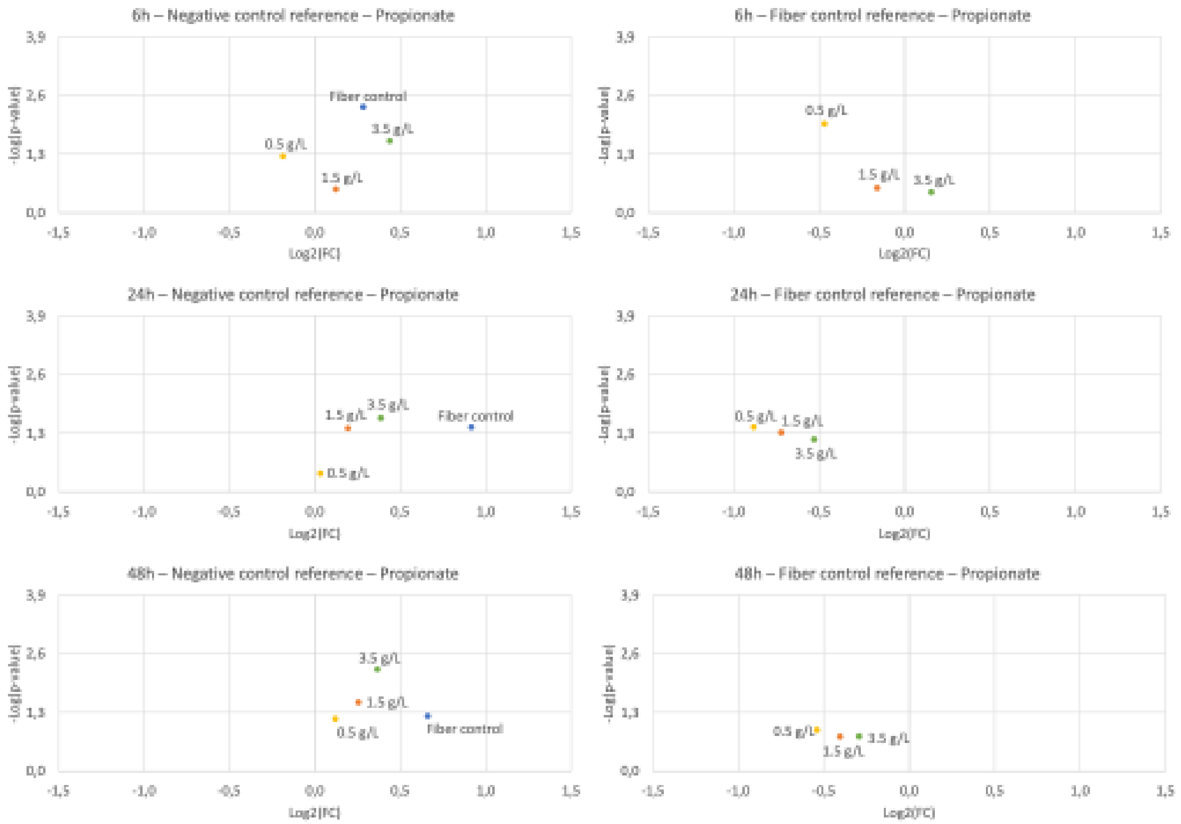

Figure S4: Volcano plot showing differences in propionate production between treatments (0.5, 1.5, 3.5 g/L seaweed extract and fiber control (3.5 g/L chicory inulin)) and negative control (left) and between treatments (0.5, 1.5, 3.5 g/L seaweed extract) and the fiber control (3.5 g/L chicory inulin) (right) after 6h, 24h and 48h of incubation across the five healthy donors. Statistical significance (-log(p-value)) is plotted in function of fold change (log2(FC)), classifying treatments into one of four categories: non-significantly lower propionate production than respective reference (bottom left), significantly lower propionate production than respective reference (top left), non-significantly higher propionate production than respective reference (bottom right) and significantly higher propionate production than respective reference (top right). The 1.3 value on the y-axis marks the level above which an effect is statistically significant compared to respective reference condition (corresponding with $p<0.05$). The zero value on the x-axis marks the separation between elevated propionate production versus respective reference condition (right) or lower propionate production than respective reference condition (left).

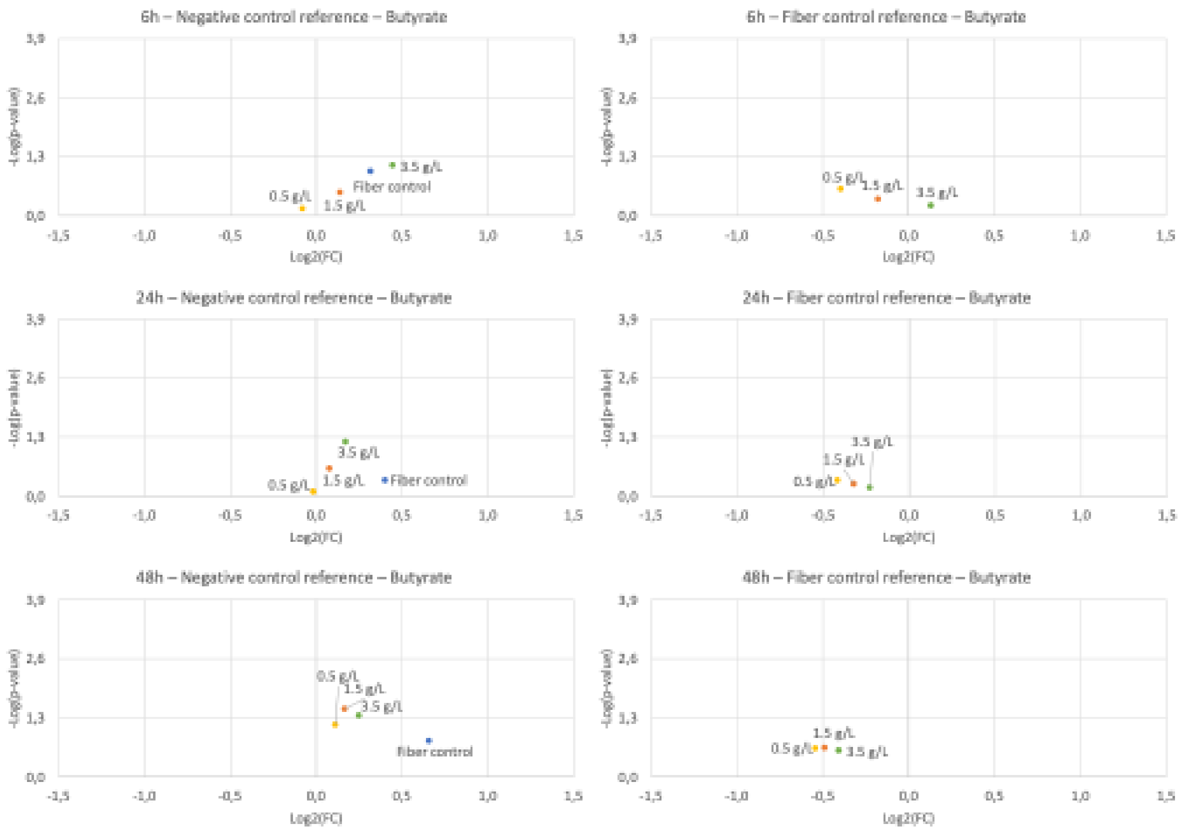

Figure S5: Volcano plot showing differences in butyrate production between treatments (0.5, 1.5, 3.5 g/L seaweed extract and fiber control (3.5 g/L chicory inulin)) and negative control (left) and between treatments (0.5, 1.5, 3.5 g/L seaweed extract) and the fiber control (3.5 g/L chicory inulin) (right) after 6h, 24h and 48h of incubation across the five healthy donors. Statistical significance (-log(p-value)) is plotted in function of fold change (log2(FC)), classifying treatments into one of four categories: non-significantly lower butyrate production than respective reference (bottom left), significantly lower butyrate production than respective reference (top left), non-significantly higher butyrate production than respective reference (bottom right) and significantly higher butyrate production than respective reference (top right). The 1.3 value on the y-axis marks the level above which an effect is statistically significant compared to respective reference condition (corresponding with $p<0.05$). The zero value on the x-axis marks the separation between elevated butyrate production versus respective reference condition (right) or lower butyrate production than respective reference condition (left).

Changes in cardiac metabolite composition

UHPLC-HRMS profile

The metabolite profile was established for 14 out of 20 targeted metabolites for which confident identification was possible based on the availability of authentic reference standards. Neither of the two additional targeted phlorotannins, Bifuhalol A and Bifuhalol B were detected above the limits of detection in any samples. A full overview of the metabolic coverage is shown in Table S1.

Table S1. Overview of the metabolic coverage as obtained for the targeted metabolites in the in vitro fluid samples. More specifically, it was indicated whether a metabolite was obtained above the methodological limit of quantification (LOQ). Green indicates detected, red indicates below LOQ.

nr	Metabolite name	Detection status
1	Trimethylamine-N-oxide (TMAO)	Green
2	Trimethylamine (TMA)	Green
3	Choline	Green
4	Carnitine	Green
5	Betaine	Green
6	N,N-Dimethylglycine (DMG)	Green
7	Leucine	Green
8	Isoleucine	Green
9	Valine	Green
10	Glycocholic acid (GCA)	Green
11	Glycochenodeoxycholic acid (GCDCA)	Red
12	Glycoursodeoxycholic acid (GUDCA)	Red
13	Taurocholic acid (TCA)	Green
14	Indole	Green
15	Indoxyl sulphuric acid	Red
16	Indole-3-propionic acid (IPA)	Green
17	S-adenosylmethionine (SAM)	Red
18	Phenylacetic acid	Red
19	p-Cresol sulphuric acid	Red
20	p-Cresol (4-methylphenol)	Green
21	Bifuhalol-type 1	Red
22	Bifuhalol-type 2	Red

Multivariate statistical analysis

In first instance, PCA-X modelling was performed to assess the natural patterning of samples and to define the analytical performance of the UHPLC-HRMS analysis. The PCA-X model was constructed based on all biological samples and the iQC samples, and was composed of three principal components, whereby the first two principal components explained 68.5% of the present X-variance. The associated PCA-X score plot (showed a narrow cluster for the iQC samples (two clusters, associated with the two types of iQC samples), indicating good analytical performance **(**Figure S6). Hotellings T2 test revealed two outliers, namely samples P1451_C_152 and P1451_C_155, for both of which no metabolites were detected above LOQ.

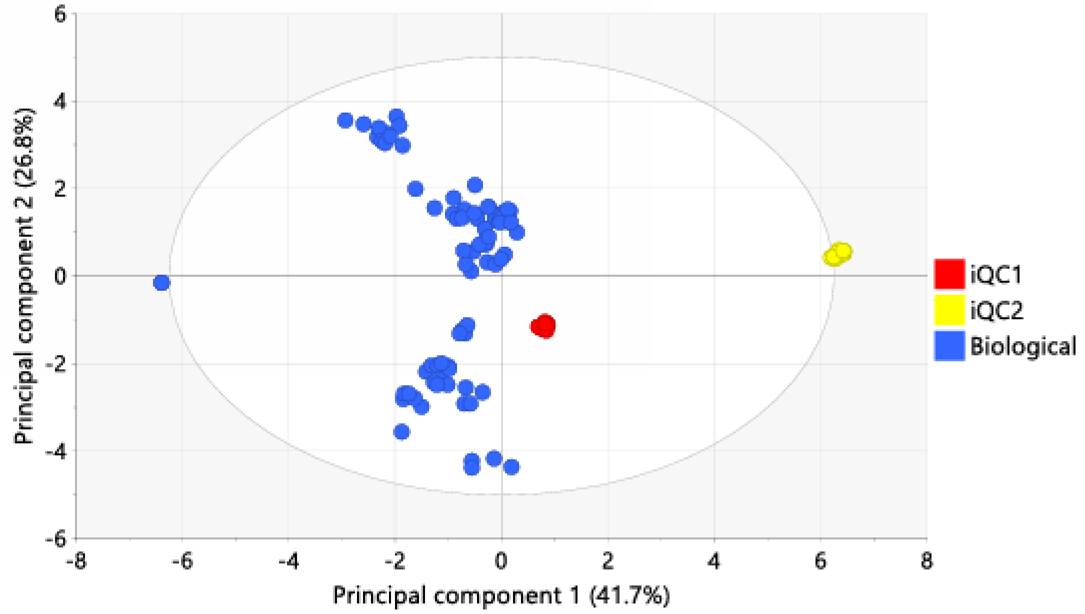

Figure S6 . PCA-X-score plot that was based on the UHPLS-HRMS data for all biological samples (n = 80) and associated iQC1 and iQC2 samples (n = 22). Two separate clusters of iQC1 and iQC2 samples were observed.

Following the initial assessment of the sample clustering, the potential major sources of biological variation were evaluated to support further data interpretation strategies. In order to investigate these sources, iQC samples were excluded from the sample set. First, differences in treatments were investigated regardless of timepoint. A PCA-X model was constructed based on all biological samples and was composed of two principal components. The associated PCA-X-plot is presented in Figure S7. No definite clusters based on treatment could be observed, though separation along the first principal component (X-variance 43.0%) as well as second principal component (X-variance 24.0%) occurred, resulting in four distinct clusters. To further investigate differences between treatments, supervised OPLS-DA modelling was performed. Comparisons of the negative control with each other treatment did not yield any valid OPLS-DA model (data not shown). Next, comparisons between the fiber control and the seaweed extract treatments were investigated. No valid OPLS-DA model was constructed for any of the comparisons (data not shown). Lastly, the comparison of seaweed extract treatments did not yield any valid OPLS-DA models (data not shown). These results indicate the different treatments, regarded over all timepoints, impose limited variation in the cardiac health metabolism. This may differ when timepoints are taking into consideration in conjunction with

treatment effects.

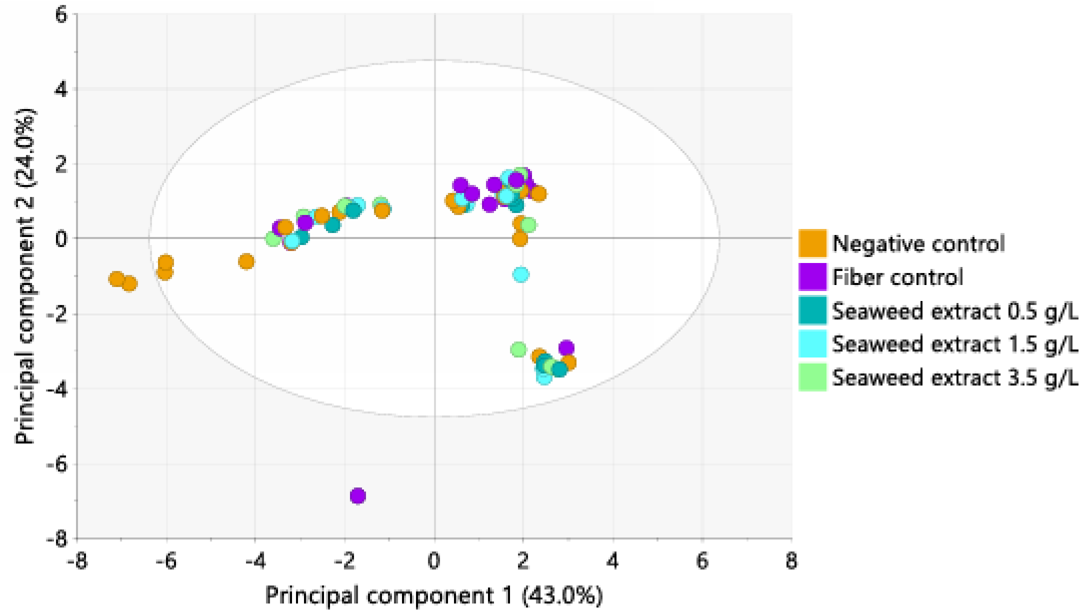

Figure S7. PCA-X-score plot that was based on the UHPLS-HRMS data for all biological samples (n = 80). Coloration was performed according to treatment.

When designating the points in the PCA-X-plot based on all biological samples to their associated timepoints, it became clear that the clusters corresponded to specific timepoints (Figure S8**).** The 0h, 6h and combined 24-48h timepoints were separated along the first principal component, with further separation of the 24h and 48h timepoint along the second principal component. **Supervised OPLS-DA modelling was able to confirm these observations, revealing significant metabolic alterations between each of the timepoints (Table S2).** These results point towards a large effect of the timepoints, which have a substantial metabolic impact on our samples.

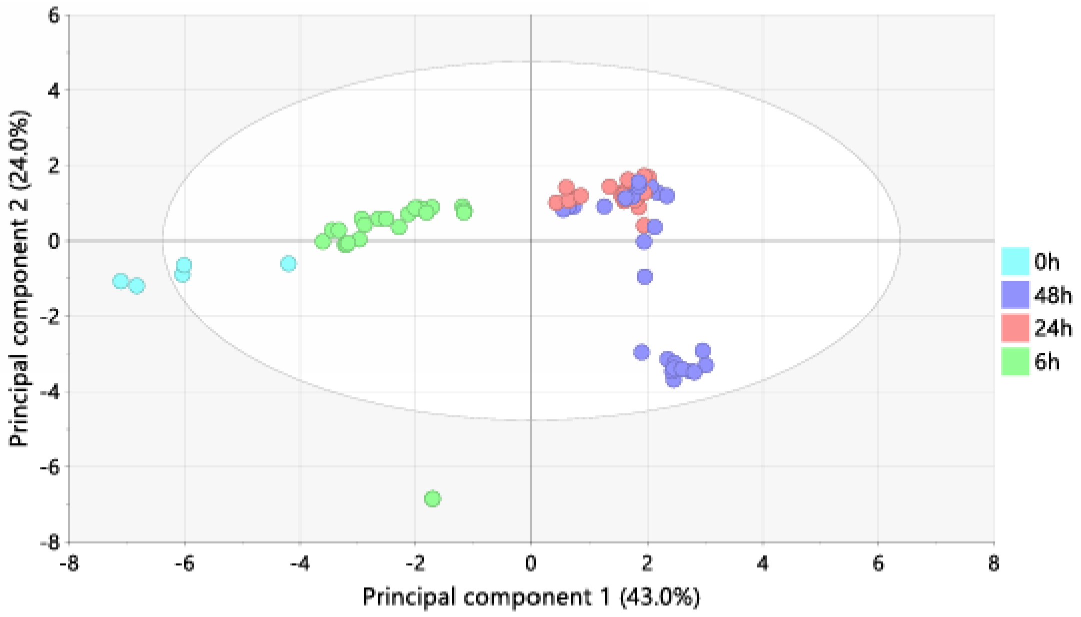

Figure S8. PCA-X-score plot that was based on the UHPLS-HRMS data for all biological samples (n = 80). Coloration was performed according to timepoints.

Table S2. OPLS-DA validation parameters for the models comparing timepoints to assess the significance of the differences by supervised modelling. Abbreviations used; tp, predictive principal component and to, orthogonal principal component.

Model (t_p + t_o)	R^2X	Q^2X	Q^2Y	Permutation test	CV-ANOVA *p*-value	Model status
0h vs 6h (1 + 4)	0.837	0.924	0.849	passed	7.41E-06	Valid
0h vs 24h (1 + 1)	0.722	0.96	0.938	passed	9.49E-15	Valid
0h vs 48h (1 + 3)	0.826	0.986	0.968	passed	5.86E-14	Valid
6h vs 24h (1 + 1)	0.689	0.949	0.941	passed	4.07E-27	Valid
6h vs 48h (1 + 2)	0.749	0.971	0.961	passed	1.59E-28	Valid
24h vs 48h (1 + 4)	0.781	0.758	0.62	passed	1.04E-05	Valid

Univariate statistical analysis

Next, based on the relative abundances of the targeted metabolites, univariate statistical analysis was applied and graphical representations of the data were created. Figure S9 shows changes in relative abundance of the metabolites of interest over time across the studied conditions. Upon assessing general trends over the 48h time frame, it was observed that overall metabolite relative abundances evolved similarly over time between the different treatments within each metabolite. TMAO, TMA, betaine, DMG, indole, IPA and p-cresol increased, whereas choline, carnitine, leucine, isoleucine, valine, GCA and TCA decreased over 48 hours, observed in all treatments. GCA and TCA were detected at the 0h timepoint, but their relative abundances dropped below the LOD from the 6h and 24h timepoint, respectively. Notably, the fiber control displayed a deviating impact in TMAO, TMA, IPA, valine, indole and p-cresol, with higher relative abundances in TMAO, valine and IPA, and a reduction in TMA, indole and p-cresol, compared to the negative control and seaweed extracts.

1.

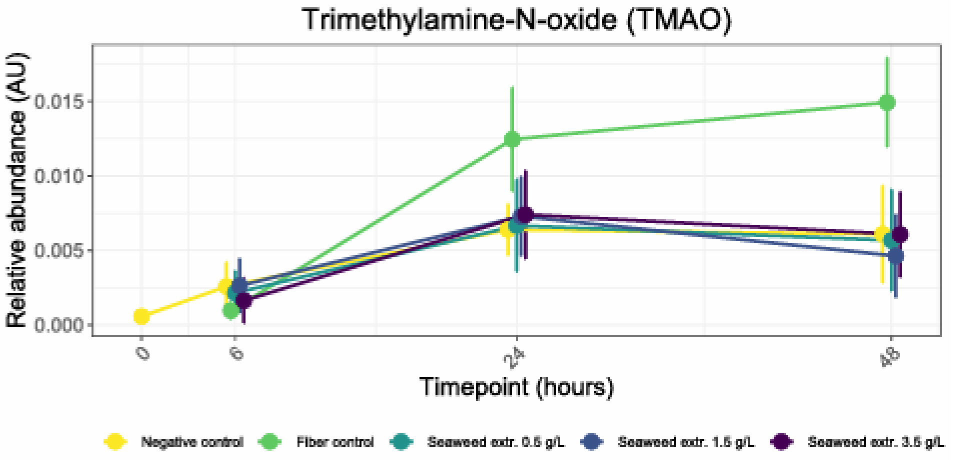
Trimethylamine-N-oxide (TMAO)
Relative abundance (AU)
0.000
0.005
0.010
0.015
0
6
24
48
Timepoint (hours)
Negative control
Fiber control
Seaweed extr. 0.5 g/L
Seaweed extr. 1.5 g/L
Seaweed extr. 3.5 g/L

2.

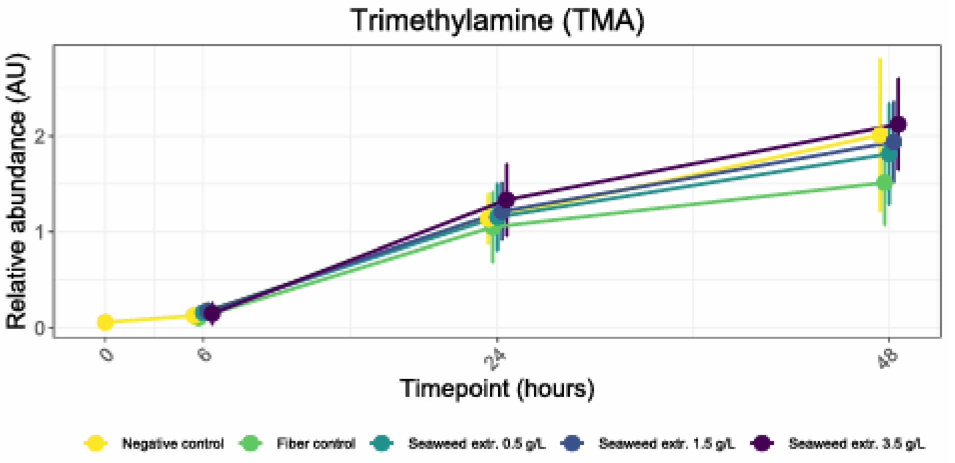
Trimethylamine (TMA)
Relative abundance (AU)
0
1
2
0
6
24
48
Timepoint (hours)
Negative control
Fiber control
Seaweed extr. 0.5 g/L
Seaweed extr. 1.5 g/L
Seaweed extr. 3.5 g/L

3.

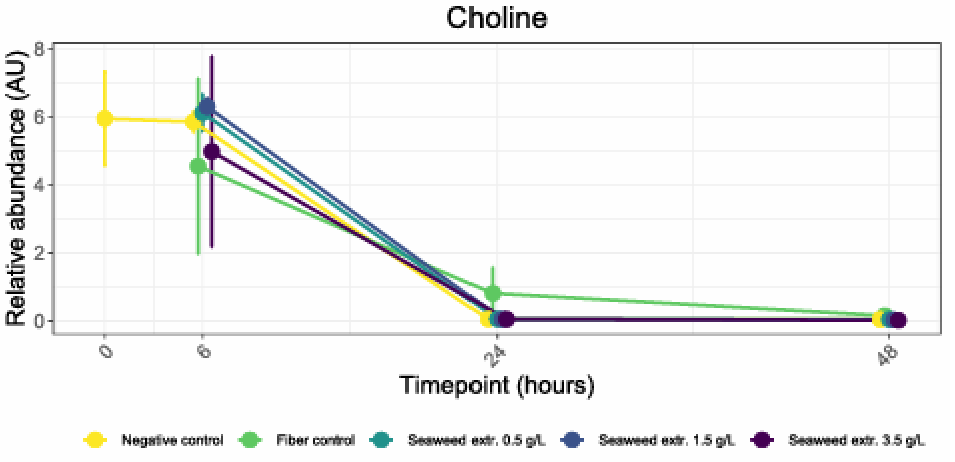
Choline
Relative abundance (AU)
0
2
4
6
8
0
6
24
48
Timepoint (hours)
Negative control
Fiber control
Seaweed extr. 0.5 g/L
Seaweed extr. 1.5 g/L
Seaweed extr. 3.5 g/L

4.

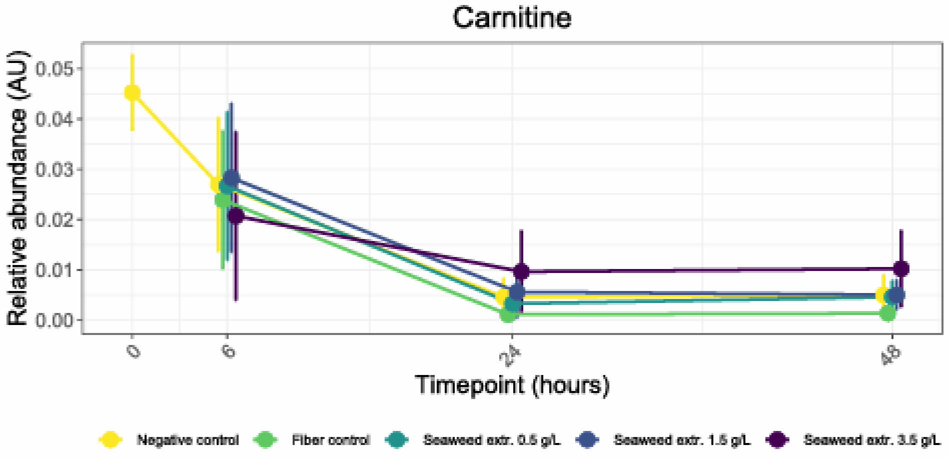
Carnitine
Relative abundance (AU)
0.05
0.04
0.03
0.02
0.01
0.00
0
6
24
48
Timepoint (hours)
Negative control
Fiber control
Seaweed extr. 0.5 g/L
Seaweed extr. 1.5 g/L
Seaweed extr. 3.5 g/L

5.

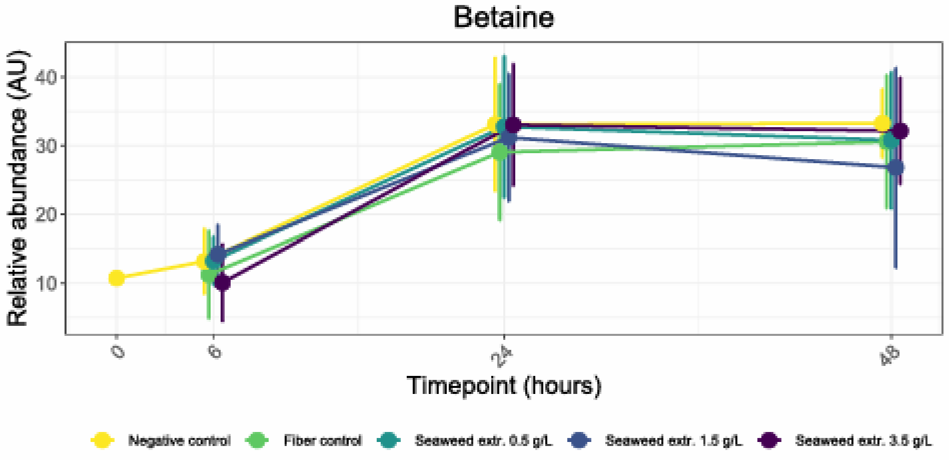
Betaine
Relative abundance (AU)
40
30
20
10
0
6
24
48
Timepoint (hours)
Negative control
Fiber control
Seaweed extr. 0.5 g/L
Seaweed extr. 1.5 g/L
Seaweed extr. 3.5 g/L

6.

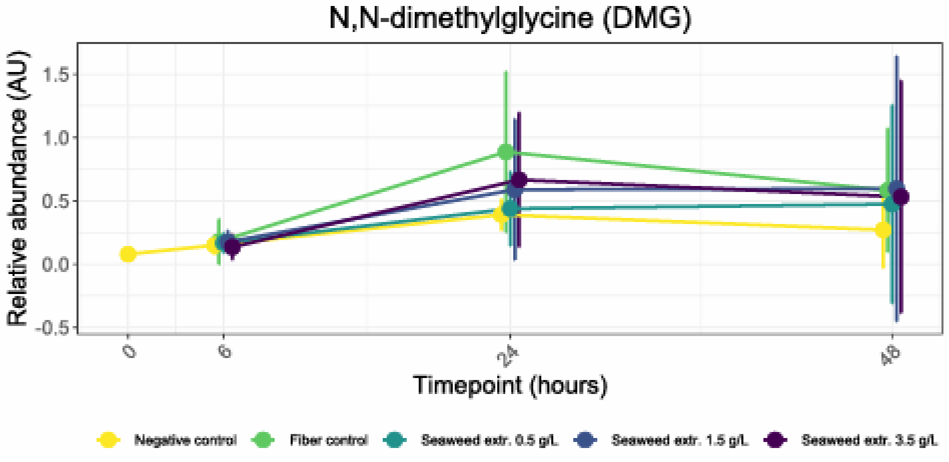
N,N-dimethylglycine (DMG)
Relative abundance (AU)
1.5
1.0
0.5
0.0
-0.5
0
6
24
48
Timepoint (hours)
Negative control
Fiber control
Seaweed extr. 0.5 g/L
Seaweed extr. 1.5 g/L
Seaweed extr. 3.5 g/L

7.

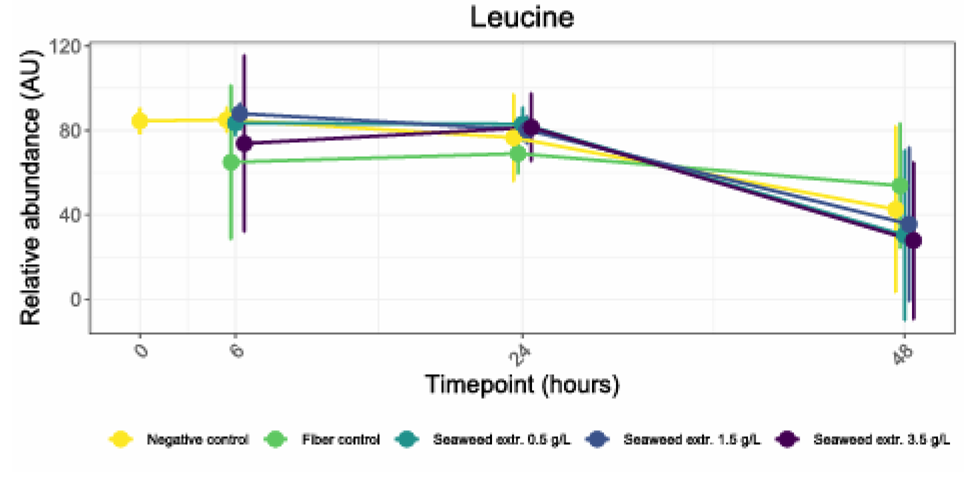
Leucine
Relative abundance (AU)
120
80
40
0
0
6
24
48
Timepoint (hours)
Negative control
Fiber control
Seaweed extr. 0.5 g/L
Seaweed extr. 1.5 g/L
Seaweed extr. 3.5 g/L

8.

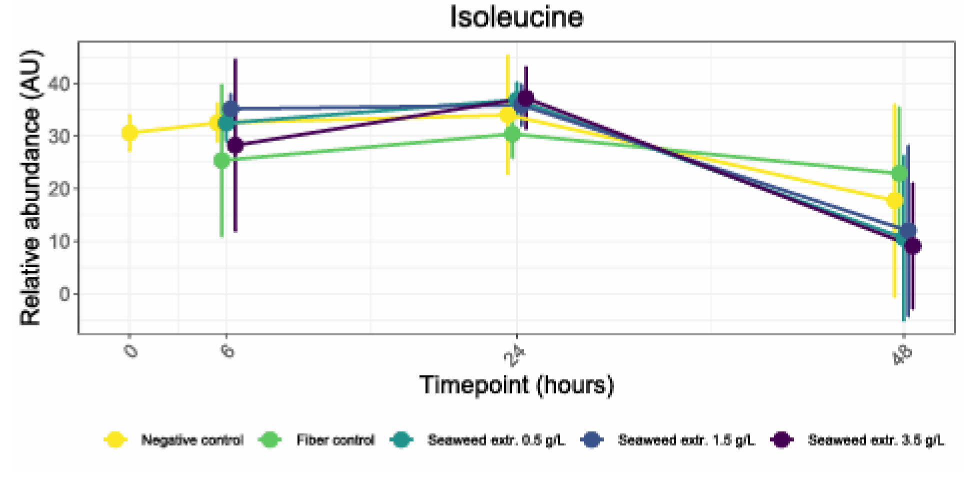
Isoleucine
Relative abundance (AU)
40
30
20
10
0
0
6
24
48
Timepoint (hours)
Negative control
Fiber control
Seaweed extr. 0.5 g/L
Seaweed extr. 1.5 g/L
Seaweed extr. 3.5 g/L

9.

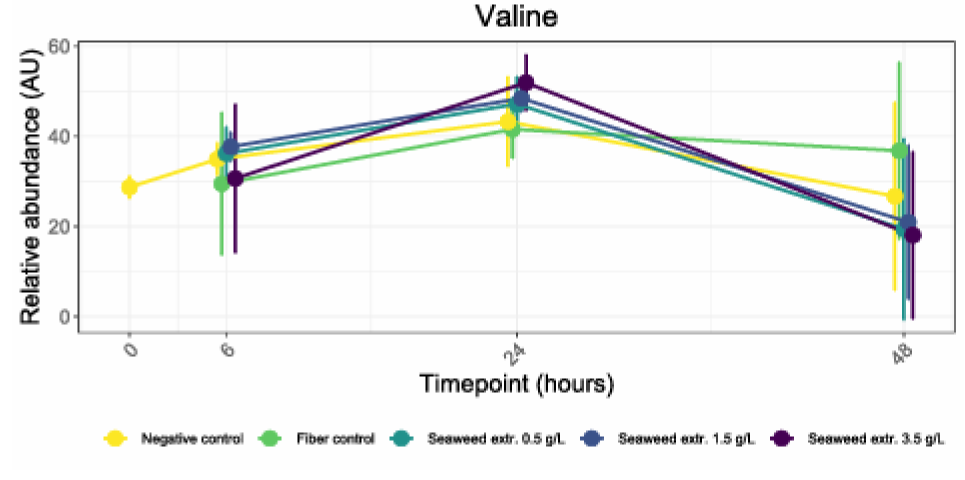
Valine
Relative abundance (AU)
60
40
20
0
0
6
24
48
Timepoint (hours)
Negative control
Fiber control
Seaweed extr. 0.5 g/L
Seaweed extr. 1.5 g/L
Seaweed extr. 3.5 g/L

10.	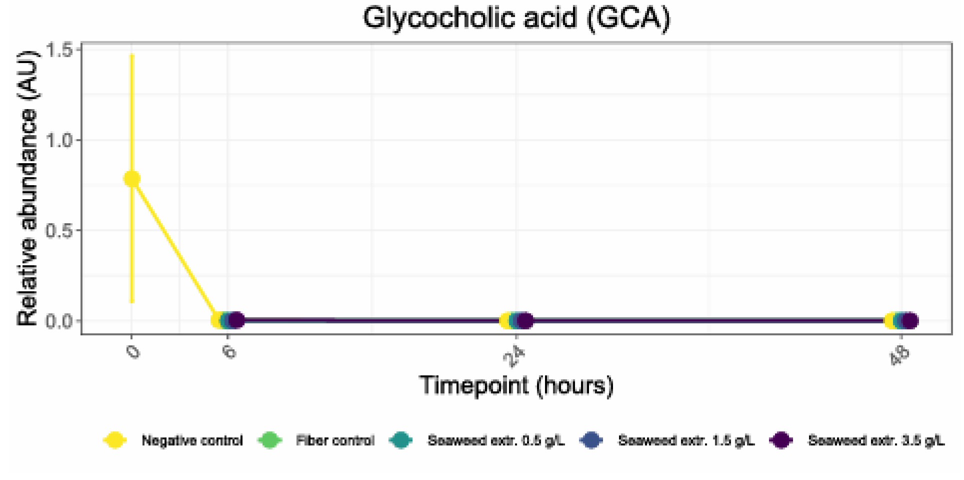 Glycocholic acid (GCA)<br>Relative abundance (AU)<br>Timepoint (hours)<br>Negative control<br>Fiber control<br>Seaweed extr. 0.5 g/L<br>Seaweed extr. 1.5 g/L<br>Seaweed extr. 3.5 g/L
13.	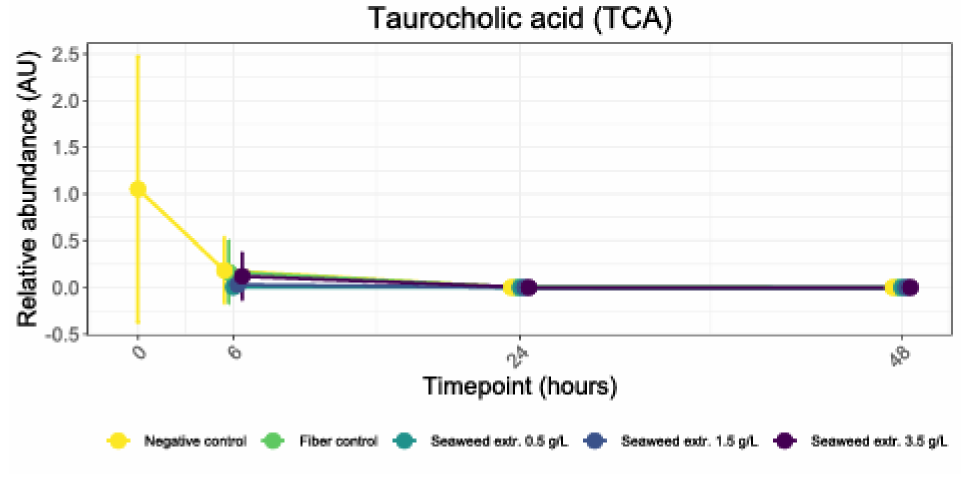 Taurocholic acid (TCA)<br>Relative abundance (AU)<br>Timepoint (hours)<br>Negative control<br>Fiber control<br>Seaweed extr. 0.5 g/L<br>Seaweed extr. 1.5 g/L<br>Seaweed extr. 3.5 g/L
14.	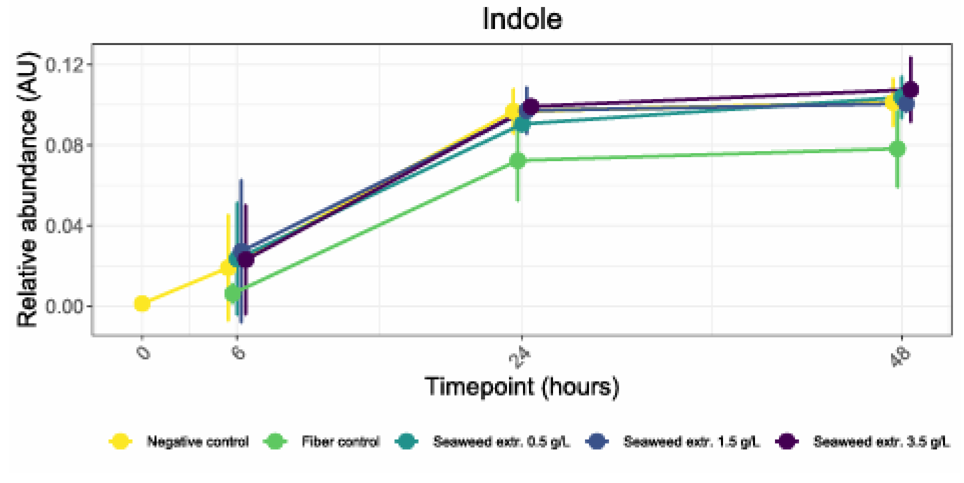 Indole<br>Relative abundance (AU)<br>Timepoint (hours)<br>Negative control<br>Fiber control<br>Seaweed extr. 0.5 g/L<br>Seaweed extr. 1.5 g/L<br>Seaweed extr. 3.5 g/L

16.

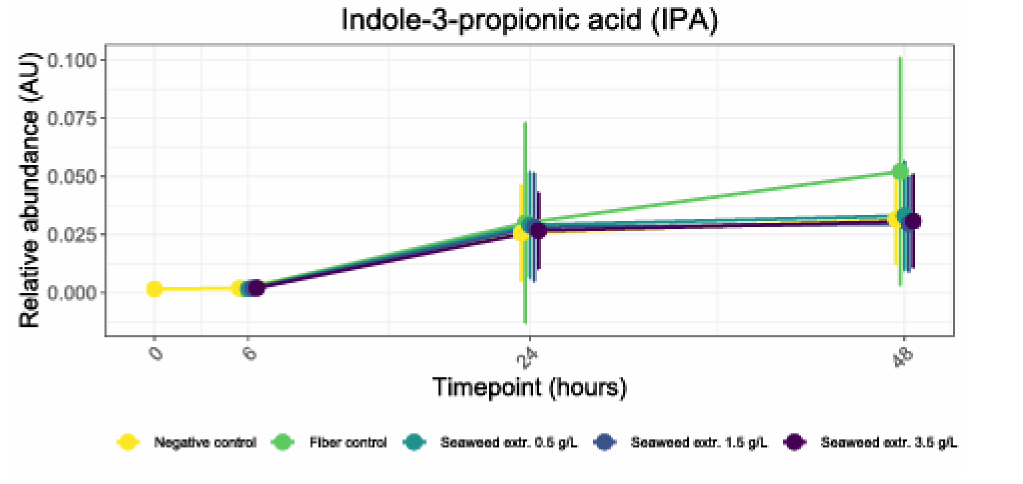

20.

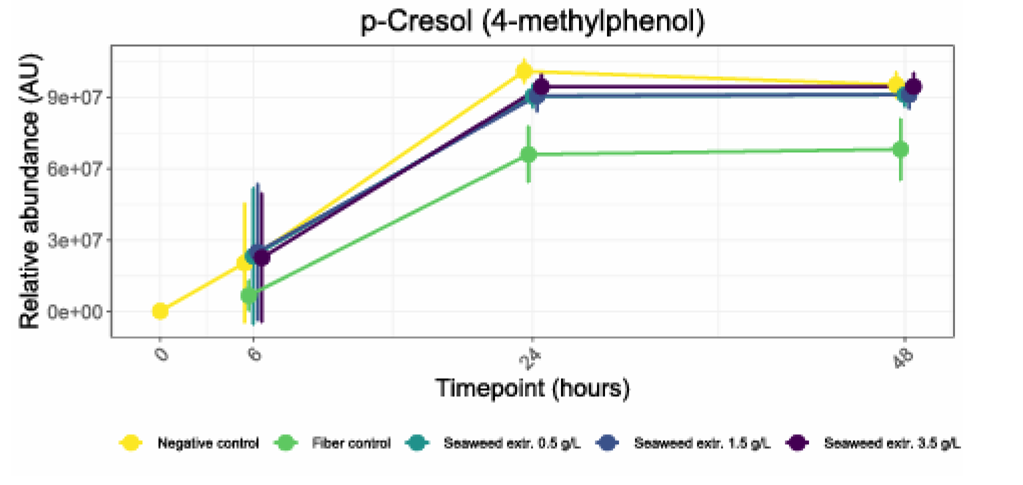

Figure S9. Changes in metabolite concentrations (relative abundance, arbitrary unit AU) between different timepoints of the inoculation (0h, 6h, 24h and 48h), per treatment condition (negative control, fiber control, seaweed extract 0.5 g/L, seaweed extract 1.5 g/L and seaweed extract 3.5 g/L). Averages are depicted over 5 replicates, as well as the standard deviation.

A representation of bar plots was constructed per metabolite, per timepoint, to investigate the differences between the negative control and the different treatments, fiber control and seaweed extracts **(**Figure S10**)**. P-values of paired t-tests used to compare the metabolite relative abundances between the negative control and other treatments are summarized in Table S3, Table S4 and Table S5. TMAO abundances increased over time in response to all treatments. However, there is a significant increase in fiber control as compared to the negative control, with TMAO abundances having more than doubled at the 48h timepoint. There was no difference in TMAO abundances between any of the seaweed extract concentrations and the negative control. Carnitine was significantly reduced in the highest seaweed concentration at the 6h timepoint.

While choline decreased over time, initially (6h timepoint) there was an increase in response to the 0.5 g/L (ns) and 1.5 g/L (significant, ***) seaweed extract concentrations. However, at the 24h and 48h timepoints abundances had dropped to levels on par with the negative control. The fiber control remained relatively high in choline abundance at these timepoints compared to the negative control, though non-significantly. Fiber control also had an influence on indole abundances over time. While indole levels of seaweed extracts did not differ significantly from

the negative control, indole was significantly reduced at the 24h and 48h timepoint in response to the fiber control. Lastly, p-Cresol displayed significant differences with the negative control after 24 and 48 hours of exposure to the fiber control and to the lowest seaweed extract concentration. The relative drop in p-cresol induced by the fiber control compared to the negative control and seaweed extract treatments was already visible at the 6h timepoint, though non-significant.

No significant changes as compared to the negative control were observed in TMA, DMG, leucine, isoleucine, GCA, TCA or IPA for any of the treatments.

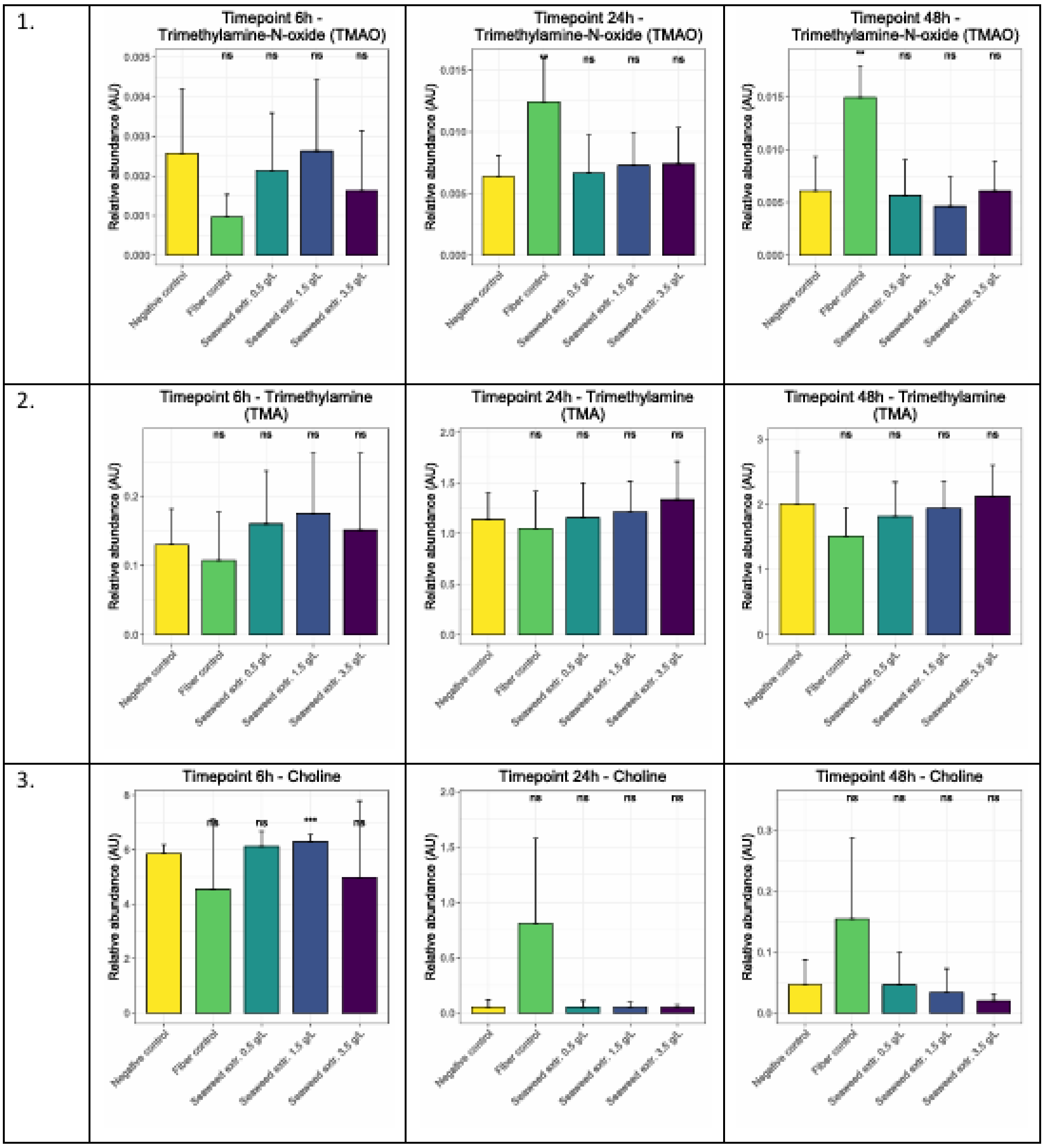

4.

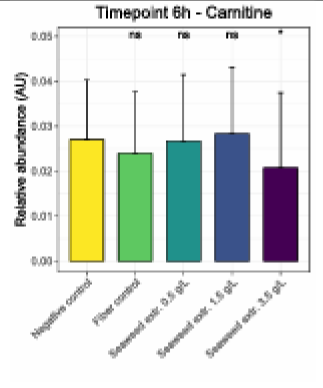
Timepoint 6h - Carnitine
Relative abundance (AU)
ns ns ns *
Negative control
Fiber control
Seaweed extr. 0.5 g/L
Seaweed extr. 1.5 g/L
Seaweed extr. 3.5 g/L

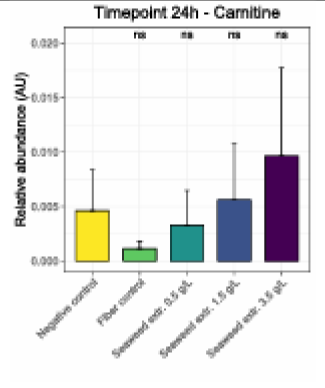
Timepoint 24h - Carnitine
Relative abundance (AU)
ns ns ns ns
Negative control
Fiber control
Seaweed extr. 0.5 g/L
Seaweed extr. 1.5 g/L
Seaweed extr. 3.5 g/L

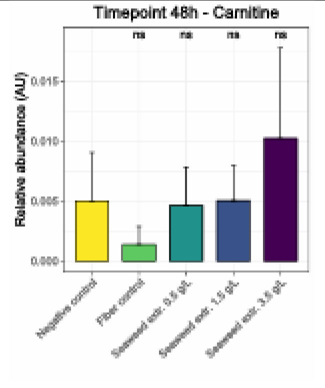
Timepoint 48h - Carnitine
Relative abundance (AU)
ns ns ns ns
Negative control
Fiber control
Seaweed extr. 0.5 g/L
Seaweed extr. 1.5 g/L
Seaweed extr. 3.5 g/L

5.

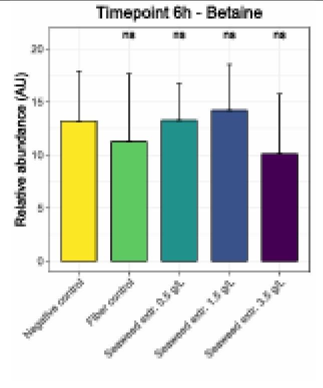
Timepoint 6h - Betaine
Relative abundance (AU)
ns ns ns ns
Negative control
Fiber control
Seaweed extr. 0.5 g/L
Seaweed extr. 1.5 g/L
Seaweed extr. 3.5 g/L

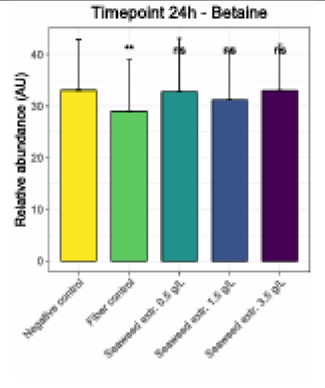
Timepoint 24h - Betaine
Relative abundance (AU)
** ns ns ns
Negative control
Fiber control
Seaweed extr. 0.5 g/L
Seaweed extr. 1.5 g/L
Seaweed extr. 3.5 g/L

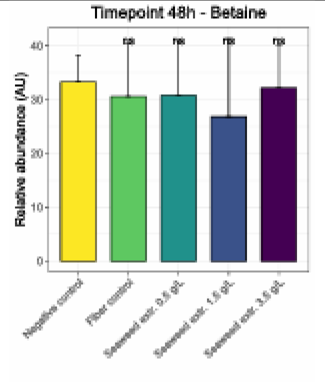
Timepoint 48h - Betaine
Relative abundance (AU)
ns ns ns ns
Negative control
Fiber control
Seaweed extr. 0.5 g/L
Seaweed extr. 1.5 g/L
Seaweed extr. 3.5 g/L

6.

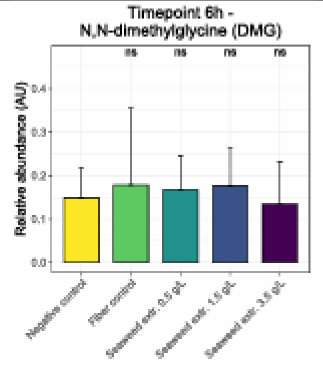
Timepoint 6h -
N,N-dimethylglycine (DMG)
Relative abundance (AU)
ns ns ns ns
Negative control
Fiber control
Seaweed extr. 0.5 g/L
Seaweed extr. 1.5 g/L
Seaweed extr. 3.5 g/L

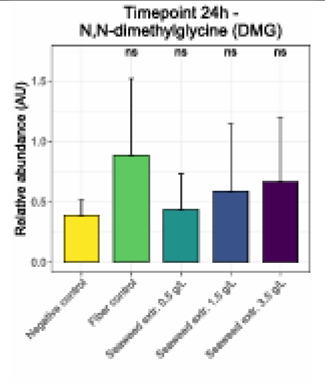
Timepoint 24h -
N,N-dimethylglycine (DMG)
Relative abundance (AU)
ns ns ns ns
Negative control
Fiber control
Seaweed extr. 0.5 g/L
Seaweed extr. 1.5 g/L
Seaweed extr. 3.5 g/L

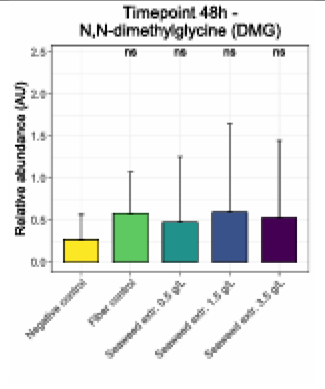
Timepoint 48h -
N,N-dimethylglycine (DMG)
Relative abundance (AU)
ns ns ns ns
Negative control
Fiber control
Seaweed extr. 0.5 g/L
Seaweed extr. 1.5 g/L
Seaweed extr. 3.5 g/L

7.

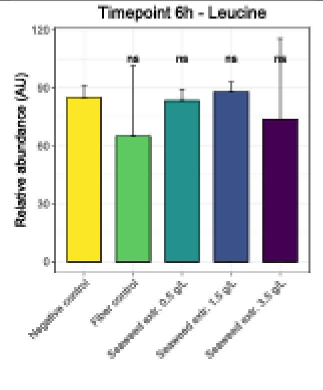
Timepoint 6h - Leucine
Relative abundance (AU)
ns ns ns ns
Negative control
Fiber control
Seaweed extr. 0.5 g/L
Seaweed extr. 1.5 g/L
Seaweed extr. 3.5 g/L

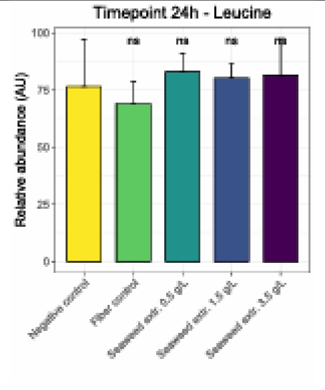
Timepoint 24h - Leucine
Relative abundance (AU)
ns ns ns ns
Negative control
Fiber control
Seaweed extr. 0.5 g/L
Seaweed extr. 1.5 g/L
Seaweed extr. 3.5 g/L

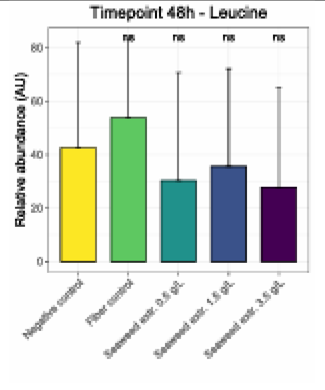
Timepoint 48h - Leucine
Relative abundance (AU)
ns ns ns ns
Negative control
Fiber control
Seaweed extr. 0.5 g/L
Seaweed extr. 1.5 g/L
Seaweed extr. 3.5 g/L

8.

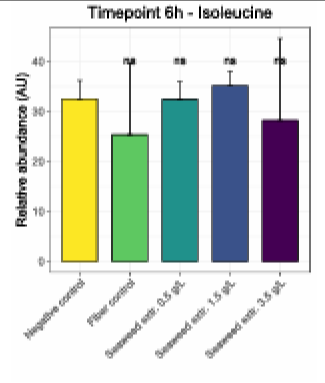
Timepoint 6h - Isoleucine
Relative abundance (AU)
Negative control
Fiber control
Seaweed extr. 0.5 g/L
Seaweed extr. 1.5 g/L
Seaweed extr. 3.5 g/L

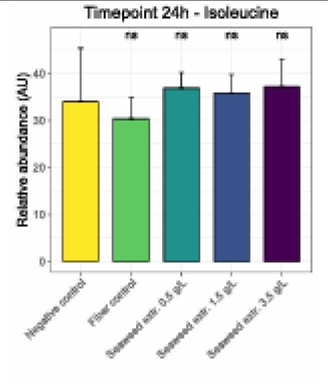
Timepoint 24h - Isoleucine
Relative abundance (AU)
Negative control
Fiber control
Seaweed extr. 0.5 g/L
Seaweed extr. 1.5 g/L
Seaweed extr. 3.5 g/L

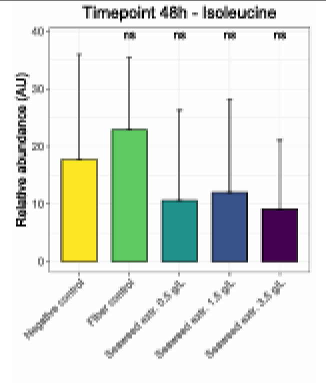
Timepoint 48h - Isoleucine
Relative abundance (AU)
Negative control
Fiber control
Seaweed extr. 0.5 g/L
Seaweed extr. 1.5 g/L
Seaweed extr. 3.5 g/L

9.

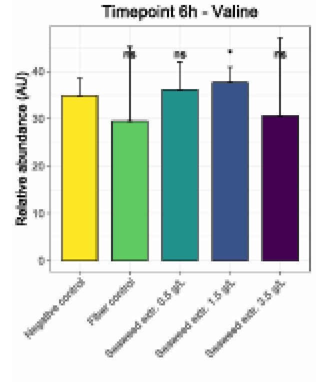
Timepoint 6h - Valine
Relative abundance (AU)
Negative control
Fiber control
Seaweed extr. 0.5 g/L
Seaweed extr. 1.5 g/L
Seaweed extr. 3.5 g/L

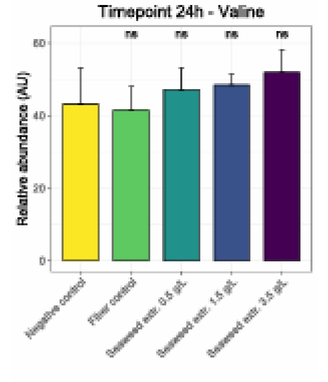
Timepoint 24h - Valine
Relative abundance (AU)
Negative control
Fiber control
Seaweed extr. 0.5 g/L
Seaweed extr. 1.5 g/L
Seaweed extr. 3.5 g/L

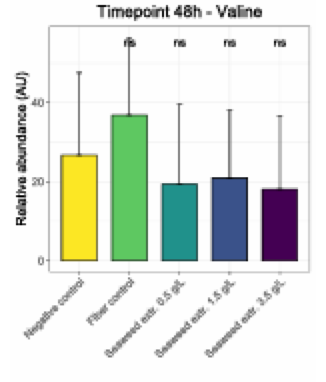
Timepoint 48h - Valine
Relative abundance (AU)
Negative control
Fiber control
Seaweed extr. 0.5 g/L
Seaweed extr. 1.5 g/L
Seaweed extr. 3.5 g/L

10.

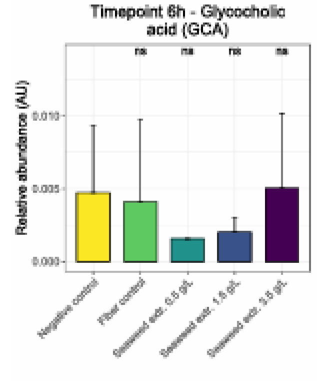
Timepoint 6h - Glycocholic acid (GCA)
Relative abundance (AU)
Negative control
Fiber control
Seaweed extr. 0.5 g/L
Seaweed extr. 1.5 g/L
Seaweed extr. 3.5 g/L

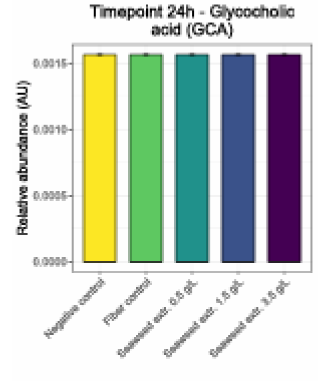
Timepoint 24h - Glycocholic acid (GCA)
Relative abundance (AU)
Negative control
Fiber control
Seaweed extr. 0.5 g/L
Seaweed extr. 1.5 g/L
Seaweed extr. 3.5 g/L

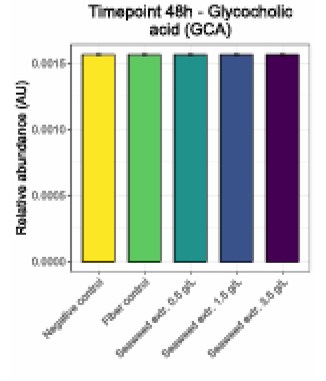
Timepoint 48h - Glycocholic acid (GCA)
Relative abundance (AU)
Negative control
Fiber control
Seaweed extr. 0.5 g/L
Seaweed extr. 1.5 g/L
Seaweed extr. 3.5 g/L

13.

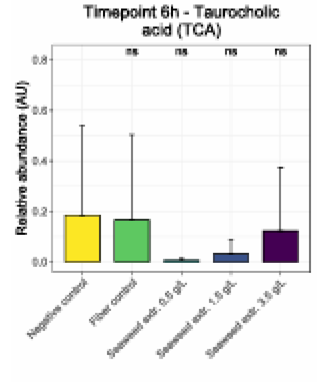
Timepoint 6h - Taurocholic acid (TCA)
Relative abundance (AU)
Negative control
Fiber control
Seaweed extr. 0.5 g/L
Seaweed extr. 1.5 g/L
Seaweed extr. 3.5 g/L

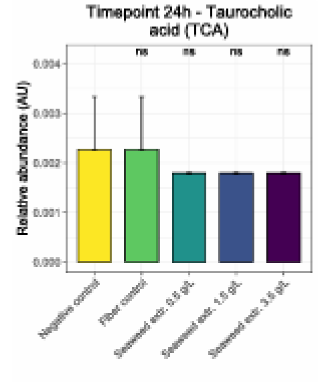
Timepoint 24h - Taurocholic acid (TCA)
Relative abundance (AU)
Negative control
Fiber control
Seaweed extr. 0.5 g/L
Seaweed extr. 1.5 g/L
Seaweed extr. 3.5 g/L

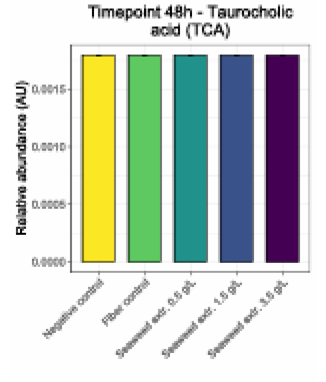
Timepoint 48h - Taurocholic acid (TCA)
Relative abundance (AU)
Negative control
Fiber control
Seaweed extr. 0.5 g/L
Seaweed extr. 1.5 g/L
Seaweed extr. 3.5 g/L

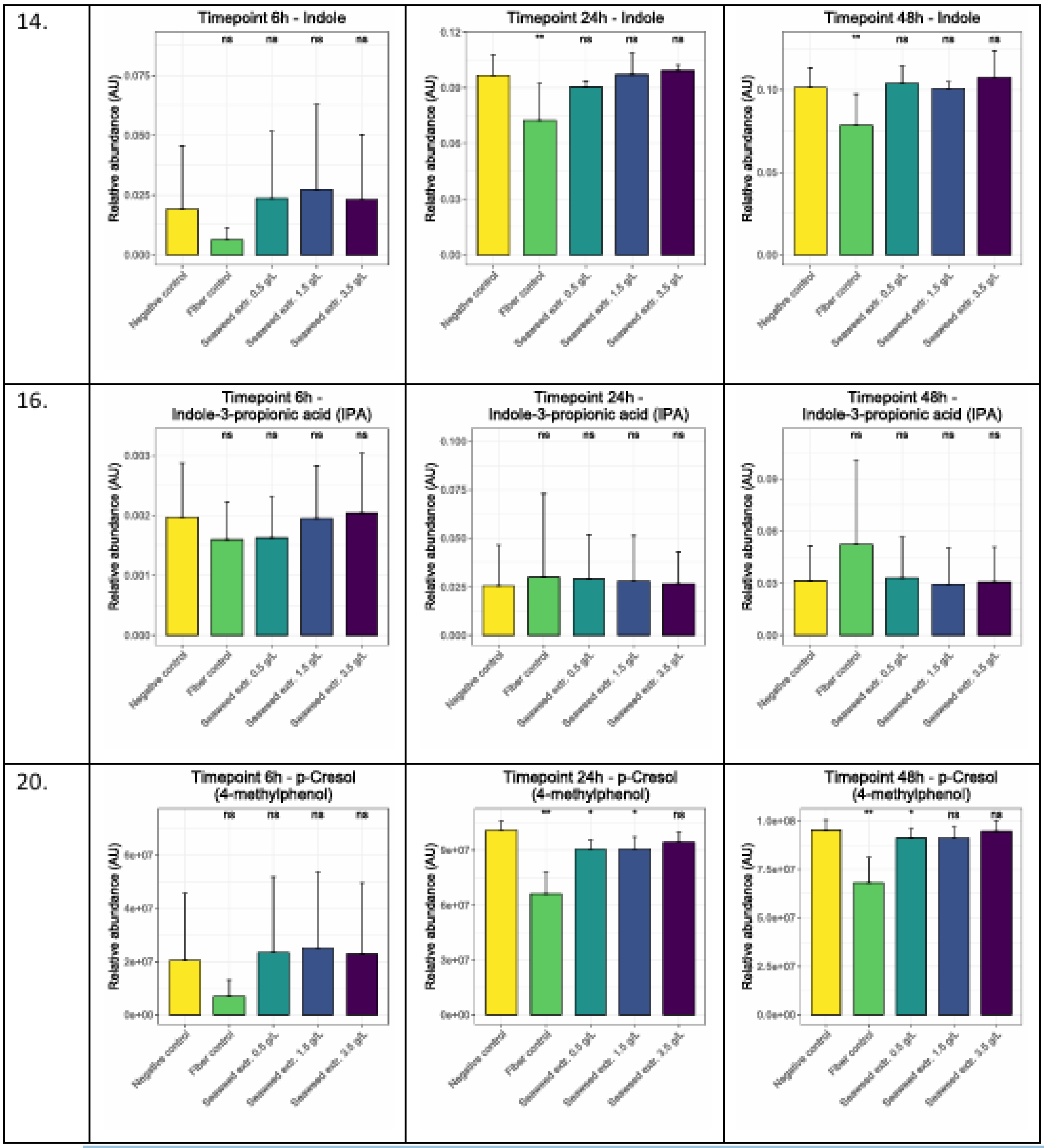

Figure S10. Bar plots of relative abundances at different timepoints (left, 6h timepoint; center, 24h timepoint; right, 48h timepoint) of each treatment condition (negative control, fiber control, seaweed extract 0.5 g/L, seaweed extract 1.5 g/L and seaweed extract 3.5 g/L). Averages are depicted over 5 replicates, as well as the standard deviation. Raw p-values from paired t-test results of comparison with the negative control are shown, with ns raw p-value > 0.05; * raw p-value ≤ 0.05; ** raw p-value ≤ 0.01; *** raw p-value ≤ 0.001.

Table S3. p-values of the paired t-tests comparing metabolite levels between the negative control and other treatments at timepoint 6h. p-values depicted in bold show significant differences. Significance cutoffs are set at ns raw p-value > 0.05; * raw p-value ≤ 0.05; *** raw p-value ≤ 0.001.

	Negative control vs	Fiber control		Seaweed extr. 0.5 g/L		Seaweed extr. 1.5 g/L		Seaweed extr. 3.5 g/L	
nr	metabolite	*p*-val.	Signif.	*p*-val.	Signif.	*p*-val.	Signif.	*p*-val.	Signif.
1	Trimethylamine-N-oxide (TMAO)	0.091	ns	0.288	ns	0.882	ns	0.224	ns
2	Trimethylamine (TMA)	0.304	ns	0.197	ns	0.086	ns	0.533	ns
3	Choline	0.346	ns	0.481	ns	**0.001**	***	0.546	ns
4	Carnitine	0.570	ns	0.928	ns	0.568	ns	**0.043**	*
5	Betaine	0.695	ns	0.954	ns	0.103	ns	0.507	ns
6	N,N-dimethylglycine (DMG)	0.765	ns	0.176	ns	0.118	ns	0.786	ns
7	Leucine	0.308	ns	0.467	ns	0.149	ns	0.595	ns
8	Isoleucine	0.306	ns	0.958	ns	0.124	ns	0.570	ns
9	Valine	0.505	ns	0.374	ns	**0.017**	*	0.609	ns
10	Glycocholic acid (GCA)	0.478	ns	0.200	ns	0.173	ns	0.676	ns
13	Taurocholic acid (TCA)	0.142	ns	0.316	ns	0.315	ns	0.261	ns
14	Indole	0.325	ns	0.083	ns	0.137	ns	0.402	ns
16	Indole-3-propionic acid (IPA)	0.298	ns	0.347	ns	0.738	ns	0.349	ns
20	p-Cresol (4-methylphenol)	0.294	ns	0.202	ns	0.115	ns	0.597	ns

Table S4. p-values of the paired t-tests comparing metabolite levels between the negative control and other treatments at timepoint 24h. p-values depicted in bold show significant differences. Significance cutoffs are set at ns raw p-value > 0.05; * raw p-value ≤ 0.05; ** raw p-value ≤ 0.01.

	Negative control vs:	Fiber control		Seaweed extr. 0.5 g/L		Seaweed extr. 1.5 g/L		Seaweed extr. 3.5 g/L	
nr	metabolite	*p*-val.	Signif.	*p*-val.	Signif.	*p*-val.	Signif.	*p*-val.	Signif.
1	Trimethylamine-N-oxide (TMAO)	**0.008**	**	0.696	ns	0.336	ns	0.272	ns
2	Trimethylamine (TMA)	0.672	ns	0.697	ns	0.208	ns	0.065	ns
3	Choline	0.103	ns	0.893	ns	0.867	ns	0.864	ns
4	Carnitine	0.093	ns	0.353	ns	0.571	ns	0.116	ns
5	Betaine	**0.002**	**	0.713	ns	0.221	ns	0.949	ns
6	N,N-dimethylglycine (DMG)	0.190	ns	0.692	ns	0.422	ns	0.237	ns
7	Leucine	0.567	ns	0.458	ns	0.650	ns	0.166	ns
8	Isoleucine	0.617	ns	0.538	ns	0.786	ns	0.564	ns
9	Valine	0.762	ns	0.401	ns	0.282	ns	0.088	ns
10	Glycocholic acid (GCA)	NA	ns	NA	ns	NA	ns	NA	ns
13	Taurocholic acid (TCA)	0.374	ns	0.374	ns	0.374	ns	0.374	ns
14	Indole	**0.009**	**	0.255	ns	0.883	ns	0.590	ns
16	Indole-3-propionic acid (IPA)	0.716	ns	0.509	ns	0.741	ns	0.892	ns
20	p-Cresol (4-methylphenol)	**0.003**	**	**0.047**	*	**0.019**	*	0.241	ns

Table S5 p-values of the paired t-tests comparing metabolite levels between the negative control and other treatments at timepoint 48h. p-values depicted in bold show significant differences. Significance cutoffs are set at ns raw p-value > 0.05; * raw p-value ≤ 0.05; ** raw p-value ≤ 0.01.

	Negative control vs:	Fiber control		Seaweed extr. 0.5 g/L		Seaweed extr. 1.5 g/L		Seaweed extr. 3.5 g/L	
nr	metabolite	*p*-val.	Signif.	*p*-val.	Signif.	*p*-val.	Signif.	*p*-val.	Signif.
1	Trimethylamine-N-oxide (TMAO)	**0.009**	**	0.361	ns	0.072	ns	0.988	ns
2	Trimethylamine (TMA)	0.219	ns	0.354	ns	0.742	ns	0.683	ns
3	Choline	0.187	ns	0.968	ns	0.332	ns	0.148	ns
4	Carnitine	0.074	ns	0.712	ns	0.994	ns	0.061	ns
5	Betaine	0.387	ns	0.468	ns	0.291	ns	0.722	ns
6	N,N-dimethylglycine (DMG)	0.054	ns	0.409	ns	0.393	ns	0.416	ns
7	Leucine	0.517	ns	0.321	ns	0.291	ns	0.392	ns
8	Isoleucine	0.533	ns	0.134	ns	0.180	ns	0.342	ns
9	Valine	0.385	ns	0.229	ns	0.199	ns	0.355	ns
10	Glycocholic acid (GCA)	NA	ns	NA	ns	NA	ns	NA	ns
13	Taurocholic acid (TCA)	NA	ns	NA	ns	NA	ns	NA	ns
14	Indole	**0.006**	**	0.758	ns	0.930	ns	0.486	ns
16	Indole-3-propionic acid (IPA)	0.284	ns	0.771	ns	0.678	ns	0.879	ns
20	p-Cresol (4-methylphenol)	**0.004**	**	**0.034**	*	0.146	ns	0.818	ns

Next, a representation of bar plots was constructed per metabolite, per timepoint, to investigate the differences between the fiber control and the different seaweed extract concentrations (Figure S11). P values of paired t-tests used to compare the metabolite relative abundances

between the fiber control and seaweed extract concentrations are summarized in Table S6, Table S7 and Table S8.

At the 6h timepoint, TMAO abundances were higher in the seaweed extracts compared to the fiber control (ns). While all treatments induced an increase in relative abundance of TMAO, this increase was significantly more pronounced in the fiber control as compared to the seaweed extracts after 24 hours, and also persisted after 48 hours. TMA on the other hand was significantly increased in the seaweed extracts compared to the fiber control at the 6h timepoint. A dose-dependent seaweed extract-induced increase remained visible after 24 and 48 hours, with the highest seaweed extract concentrations causing the highest increase. These increases however were non-significant in the 0.5 g/L and 1.5 g/L concentration ranges. Similar observations were made for the carnitine relative abundances, with a dose-dependent increase at the 24h timepoint (ns) and at the 48h timepoint (significant, 0.5 g/L and 1.5 g/L seaweed extracts), compared to the fiber control. Leucine abundances were increased at the 6h timepoint (3.5 g/L, significant) and 24h timepoint (0.5 g/L, significant) compared to the fiber control. After 48 hours however this increase was no longer visible, with fiber control leucine levels being higher than any of the seaweed extract concentrations. A similar observation was made for valine, where there was an apparent dose-dependent increase induced by the seaweed extracts compared to the fiber control after 24 hours, with reduced levels in the seaweed extracts at the 48h timepoint.

Both indole and p-cresol were increased compared to the fiber control at each timepoint. Moreover, all seaweed extract concentrations caused a significant increase at the 24h and 48h timepoints. For indole the increase was most apparent in the highest seaweed extract concentration. Lastly, choline abundances dropped sharply at the 24h timepoint in all seaweed extract concentrations compared to the fiber control and remained reduced at the 48h timepoint, though this reduction was non-significant.

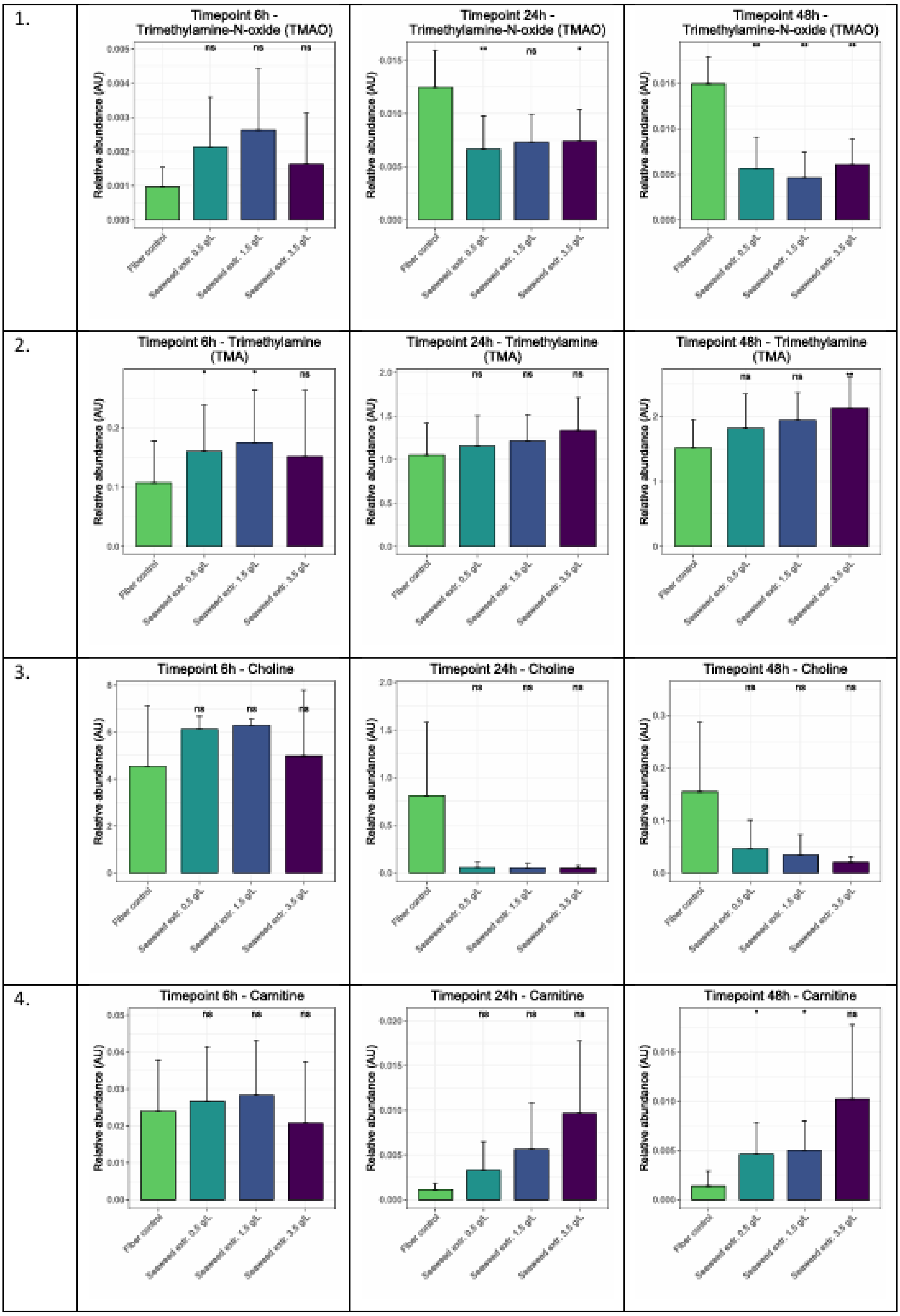
1.
Timepoint 6h - Trimethylamine-N-oxide (TMAO)
Timepoint 24h - Trimethylamine-N-oxide (TMAO)
Timepoint 48h - Trimethylamine-N-oxide (TMAO)
2.
Timepoint 6h - Trimethylamine (TMA)
Timepoint 24h - Trimethylamine (TMA)
Timepoint 48h - Trimethylamine (TMA)
3.
Timepoint 6h - Choline
Timepoint 24h - Choline
Timepoint 48h - Choline
4.
Timepoint 6h - Carnitine
Timepoint 24h - Carnitine
Timepoint 48h - Carnitine
Relative abundance (AU)
Fiber control
Seaweed extr. 0.5 g/L
Seaweed extr. 1.5 g/L
Seaweed extr. 3.5 g/L

5.

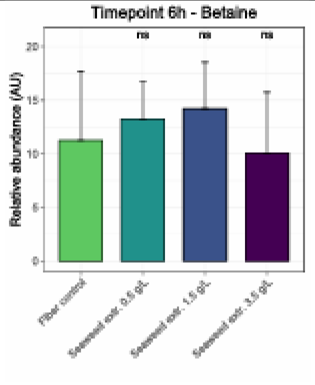
Timepoint 6h - Betaine
Relative abundance (AU)
Fiber control
Seaweed extr. 0.5 g/L
Seaweed extr. 1.5 g/L
Seaweed extr. 3.5 g/L

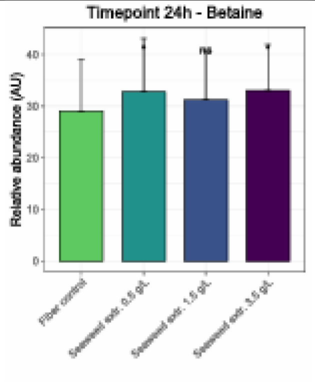
Timepoint 24h - Betaine
Relative abundance (AU)
Fiber control
Seaweed extr. 0.5 g/L
Seaweed extr. 1.5 g/L
Seaweed extr. 3.5 g/L

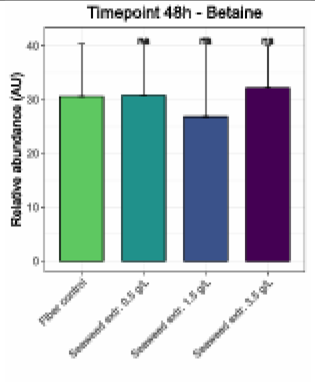
Timepoint 48h - Betaine
Relative abundance (AU)
Fiber control
Seaweed extr. 0.5 g/L
Seaweed extr. 1.5 g/L
Seaweed extr. 3.5 g/L

6.

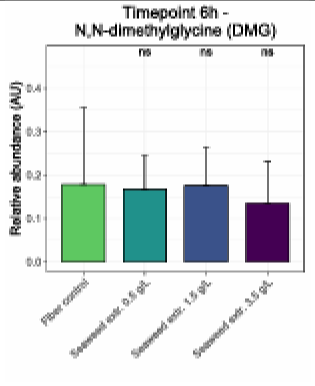
Timepoint 6h -
N,N-dimethylglycine (DMG)
Relative abundance (AU)
Fiber control
Seaweed extr. 0.5 g/L
Seaweed extr. 1.5 g/L
Seaweed extr. 3.5 g/L

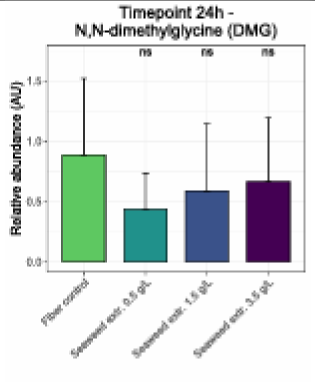
Timepoint 24h -
N,N-dimethylglycine (DMG)
Relative abundance (AU)
Fiber control
Seaweed extr. 0.5 g/L
Seaweed extr. 1.5 g/L
Seaweed extr. 3.5 g/L

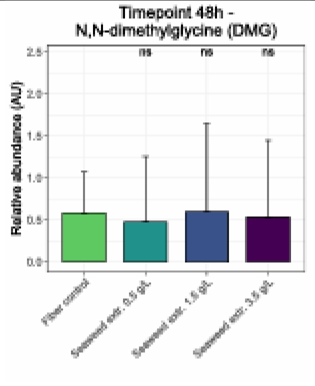
Timepoint 48h -
N,N-dimethylglycine (DMG)
Relative abundance (AU)
Fiber control
Seaweed extr. 0.5 g/L
Seaweed extr. 1.5 g/L
Seaweed extr. 3.5 g/L

7.

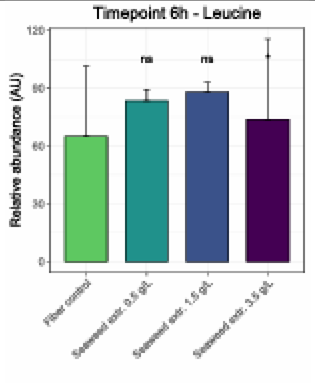
Timepoint 6h - Leucine
Relative abundance (AU)
Fiber control
Seaweed extr. 0.5 g/L
Seaweed extr. 1.5 g/L
Seaweed extr. 3.5 g/L

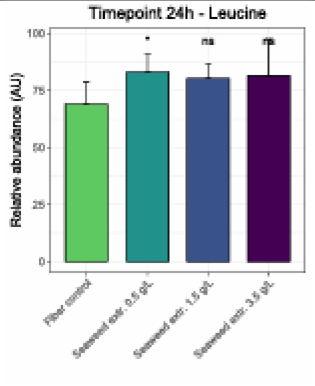
Timepoint 24h - Leucine
Relative abundance (AU)
Fiber control
Seaweed extr. 0.5 g/L
Seaweed extr. 1.5 g/L
Seaweed extr. 3.5 g/L

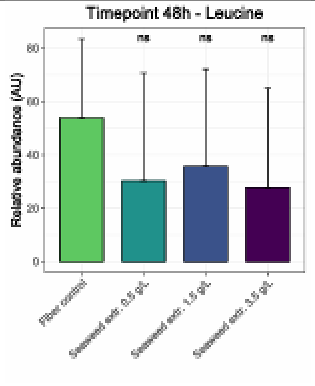
Timepoint 48h - Leucine
Relative abundance (AU)
Fiber control
Seaweed extr. 0.5 g/L
Seaweed extr. 1.5 g/L
Seaweed extr. 3.5 g/L

8.

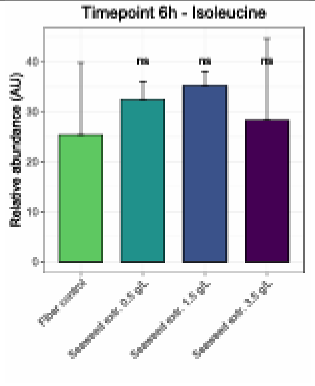
Timepoint 6h - Isoleucine
Relative abundance (AU)
Fiber control
Seaweed extr. 0.5 g/L
Seaweed extr. 1.5 g/L
Seaweed extr. 3.5 g/L

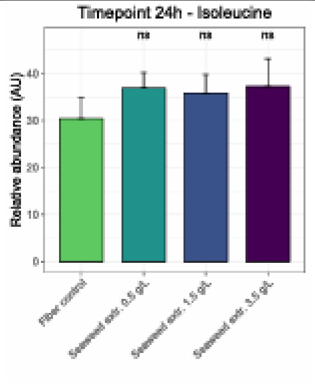
Timepoint 24h - Isoleucine
Relative abundance (AU)
Fiber control
Seaweed extr. 0.5 g/L
Seaweed extr. 1.5 g/L
Seaweed extr. 3.5 g/L

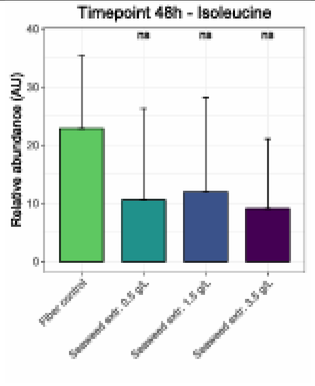
Timepoint 48h - Isoleucine
Relative abundance (AU)
Fiber control
Seaweed extr. 0.5 g/L
Seaweed extr. 1.5 g/L
Seaweed extr. 3.5 g/L

9.	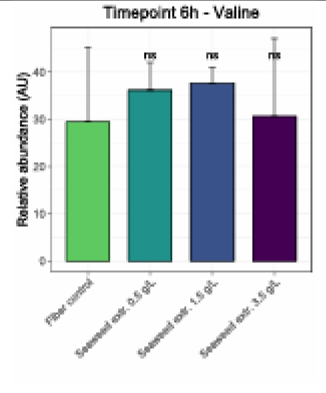 Timepoint 6h - Valine<br>Relative abundance (AU)<br>Fiber control<br>Seaweed extr. 0.5 g/L<br>Seaweed extr. 1.5 g/L<br>Seaweed extr. 3.5 g/L	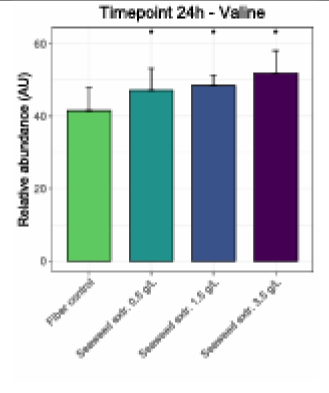 Timepoint 24h - Valine<br>Relative abundance (AU)<br>Fiber control<br>Seaweed extr. 0.5 g/L<br>Seaweed extr. 1.5 g/L<br>Seaweed extr. 3.5 g/L	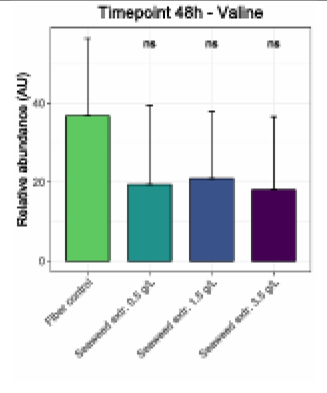 Timepoint 48h - Valine<br>Relative abundance (AU)<br>Fiber control<br>Seaweed extr. 0.5 g/L<br>Seaweed extr. 1.5 g/L<br>Seaweed extr. 3.5 g/L
10.	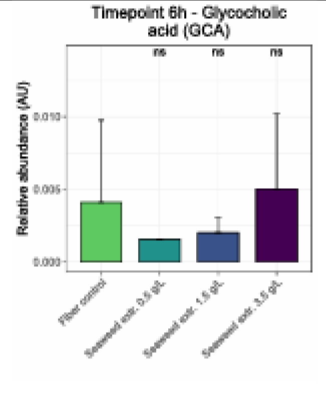 Timepoint 6h - Glycocholic acid (GCA)<br>Relative abundance (AU)<br>Fiber control<br>Seaweed extr. 0.5 g/L<br>Seaweed extr. 1.5 g/L<br>Seaweed extr. 3.5 g/L	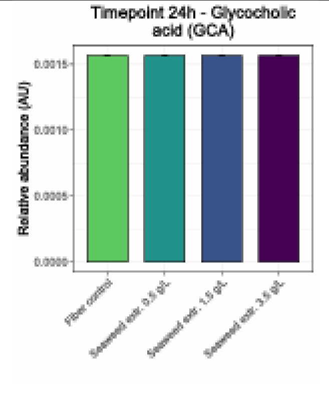 Timepoint 24h - Glycocholic acid (GCA)<br>Relative abundance (AU)<br>Fiber control<br>Seaweed extr. 0.5 g/L<br>Seaweed extr. 1.5 g/L<br>Seaweed extr. 3.5 g/L	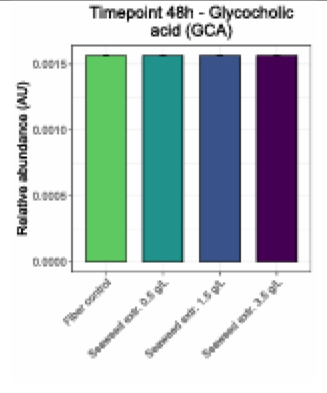 Timepoint 48h - Glycocholic acid (GCA)<br>Relative abundance (AU)<br>Fiber control<br>Seaweed extr. 0.5 g/L<br>Seaweed extr. 1.5 g/L<br>Seaweed extr. 3.5 g/L
13.	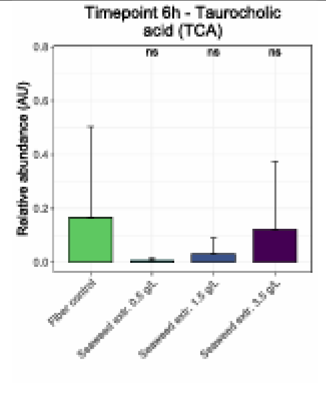 Timepoint 6h - Taurocholic acid (TCA)<br>Relative abundance (AU)<br>Fiber control<br>Seaweed extr. 0.5 g/L<br>Seaweed extr. 1.5 g/L<br>Seaweed extr. 3.5 g/L	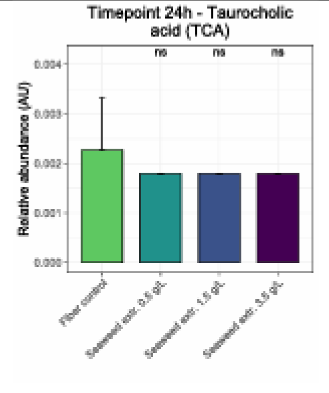 Timepoint 24h - Taurocholic acid (TCA)<br>Relative abundance (AU)<br>Fiber control<br>Seaweed extr. 0.5 g/L<br>Seaweed extr. 1.5 g/L<br>Seaweed extr. 3.5 g/L	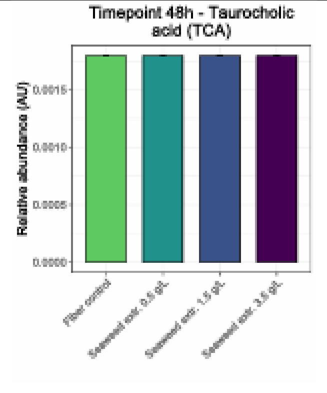 Timepoint 48h - Taurocholic acid (TCA)<br>Relative abundance (AU)<br>Fiber control<br>Seaweed extr. 0.5 g/L<br>Seaweed extr. 1.5 g/L<br>Seaweed extr. 3.5 g/L
14.	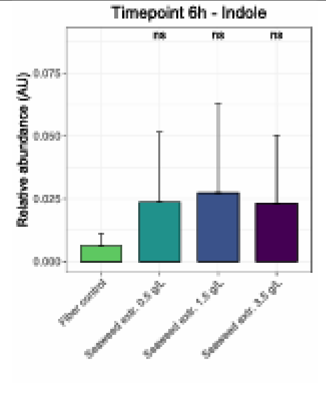 Timepoint 6h - Indole<br>Relative abundance (AU)<br>Fiber control<br>Seaweed extr. 0.5 g/L<br>Seaweed extr. 1.5 g/L<br>Seaweed extr. 3.5 g/L	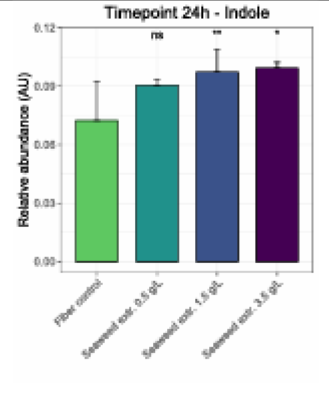 Timepoint 24h - Indole<br>Relative abundance (AU)<br>Fiber control<br>Seaweed extr. 0.5 g/L<br>Seaweed extr. 1.5 g/L<br>Seaweed extr. 3.5 g/L	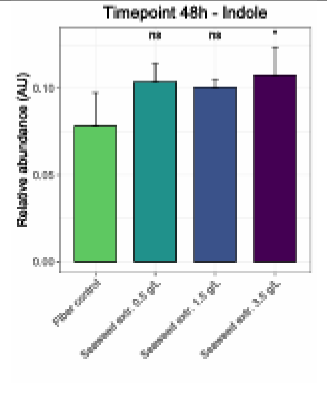 Timepoint 48h - Indole<br>Relative abundance (AU)<br>Fiber control<br>Seaweed extr. 0.5 g/L<br>Seaweed extr. 1.5 g/L<br>Seaweed extr. 3.5 g/L

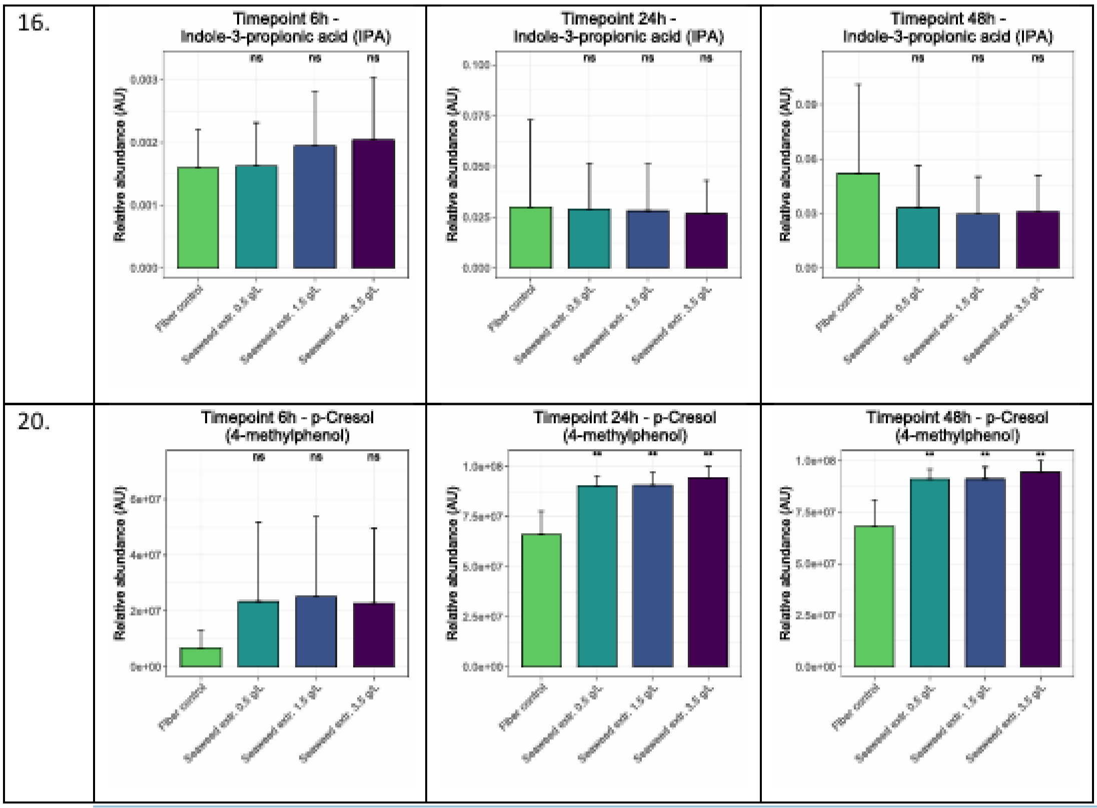

Figure S11. Bar plots of relative abundances at different timepoints (left, 6h timepoint; center, 24h timepoint; right, 48h timepoint) of treatment conditions: fiber control, seaweed extract 0.5 g/L, seaweed extract 1.5 g/L and seaweed extract 3.5 g/L. Averages are depicted over 5 replicates, as well as the standard deviation. Raw p-values from paired t-test results of comparison with the fiber control are shown, with ns raw p-value > 0.05; * raw p-value ≤ 0.05; ** raw p-value ≤ 0.01; *** raw p-value ≤ 0.001.

Table S6 p-values of the paired t-tests comparing metabolite levels between the fiber control and seaweed extracts at timepoint 6h. p-values depicted in bold show significant differences. Significance cutoffs are set at ns raw p-value > 0.05; * raw p-value ≤ 0.05.

	Fiber control vs	Seaweed extr. 0.5 g/L		Seaweed extr. 1.5 g/L		Seaweed extr. 3.5 g/L	
nr	metabolite	*p*-val.	Signif.	*p*-val.	Signif.	*p*-val.	Signif.
1	Trimethylamine-N-oxide (TMAO)	0.182	ns	0.121	ns	0.326	ns
2	Trimethylamine (TMA)	**0.025**	*	**0.016**	*	0.098	ns
3	Choline	0.283	ns	0.215	ns	0.293	ns
4	Carnitine	0.611	ns	0.551	ns	0.546	ns
5	Betaine	0.628	ns	0.532	ns	0.296	ns
6	N,N-dimethylglycine (DMG)	0.909	ns	0.978	ns	0.593	ns
7	Leucine	0.374	ns	0.254	ns	**0.030**	*
8	Isoleucine	0.326	ns	0.244	ns	0.087	ns
9	Valine	0.475	ns	0.351	ns	0.128	ns
10	Glycocholic acid (GCA)	0.374	ns	0.374	ns	0.496	ns
13	Taurocholic acid (TCA)	0.337	ns	0.340	ns	0.316	ns
14	Indole	0.223	ns	0.240	ns	0.207	ns
16	Indole-3-propionic acid (IPA)	0.950	ns	0.382	ns	0.236	ns
20	p-Cresol (4-methylphenol)	0.257	ns	0.216	ns	0.229	ns

Table S7 p-values of the paired t-tests comparing metabolite levels between the fiber control and seaweed extracts at timepoint 24h. p-values depicted in bold show significant differences. Significance cutoffs are set at ns raw p-value > 0.05; * raw p-value ≤ 0.05; ** raw p-value ≤ 0.01.

	Fiber control vs	Seaweed extr. 0.5 g/L		Seaweed extr. 1.5 g/L		Seaweed extr. 3.5 g/L	
nr	metabolite	*p*-val.	Signif.	*p*-val.	Signif.	*p*-val.	Signif.
1	Trimethylamine-N-oxide (TMAO)	**0.005**	**	0.052	ns	**0.035**	*
2	Trimethylamine (TMA)	0.619	ns	0.386	ns	0.179	ns
3	Choline	0.102	ns	0.099	ns	0.089	ns
4	Carnitine	0.142	ns	0.111	ns	0.081	ns
5	Betaine	**0.030**	*	0.194	ns	**0.025**	*
6	N,N-dimethylglycine (DMG)	0.247	ns	0.509	ns	0.641	ns
7	Leucine	**0.049**	*	0.100	ns	0.268	ns
8	Isoleucine	0.079	ns	0.071	ns	0.134	ns
9	Valine	**0.027**	*	**0.015**	*	**0.016**	*
10	Glycocholic acid (GCA)	NA	ns	NA	ns	NA	ns
13	Taurocholic acid (TCA)	0.374	ns	0.374	ns	0.374	ns
14	Indole	0.104	ns	**0.005**	**	**0.026**	*
16	Indole-3-propionic acid (IPA)	0.948	ns	0.913	ns	0.858	ns
20	p-Cresol (4-methylphenol)	**0.003**	**	**0.003**	**	**0.007**	**

Table S8. p-values of the paired t-tests comparing metabolite levels between the fiber control and seaweed extracts at timepoint 48h. p-values depicted in bold show significant differences. Significance cutoffs are set at ns raw p-value > 0.05; * raw p-value ≤ 0.05; ** raw p-value ≤ 0.01.

	Fiber control vs	Seaweed extr. 0.5 g/L		Seaweed extr. 1.5 g/L		Seaweed extr. 3.5 g/L	
nr	metabolite	*p*-val.	Signif.	*p*-val.	Signif.	*p*-val.	Signif.
1	Trimethylamine-N-oxide (TMAO)	**0.006**	**	**0.002**	**	**0.005**	**
2	Trimethylamine (TMA)	0.235	ns	0.085	ns	**0.004**	**
3	Choline	0.216	ns	0.153	ns	0.088	ns
4	Carnitine	**0.029**	*	**0.011**	*	0.057	ns
5	Betaine	0.967	ns	0.636	ns	0.787	ns
6	N,N-dimethylglycine (DMG)	0.559	ns	0.965	ns	0.828	ns
7	Leucine	0.306	ns	0.331	ns	0.170	ns
8	Isoleucine	0.193	ns	0.233	ns	0.065	ns
9	Valine	0.236	ns	0.206	ns	0.072	ns
10	Glycocholic acid (GCA)	NA	ns	NA	ns	NA	ns
13	Taurocholic acid (TCA)	NA	ns	NA	ns	NA	ns
14	Indole	0.068	ns	0.094	ns	**0.027**	*
16	Indole-3-propionic acid (IPA)	0.382	ns	0.329	ns	0.364	ns
20	p-Cresol (4-methylphenol)	**0.009**	**	**0.007**	**	**0.007**	**

Finally, a representation of bar plots was constructed per metabolite, per timepoint, to investigate the differences between the different seaweed extract concentrations (Figure S12). P-values of paired t tests used to compare the metabolite relative abundances between the seaweed extract concentrations are summarized in Table S9, Table S10 and Table S11. As this report focusses on differences between the two controls and the seaweed extracts, differences between seaweed extract concentrations will not be discussed in-depth.

1.	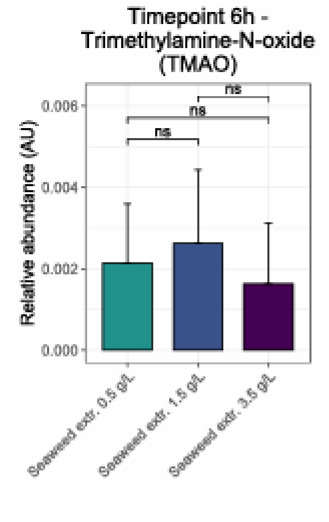 Timepoint 6h - Trimethylamine-N-oxide (TMAO)<br>Relative abundance (AU)<br>Seaweed extr. 0.5 g/L<br>Seaweed extr. 1.5 g/L<br>Seaweed extr. 3.5 g/L	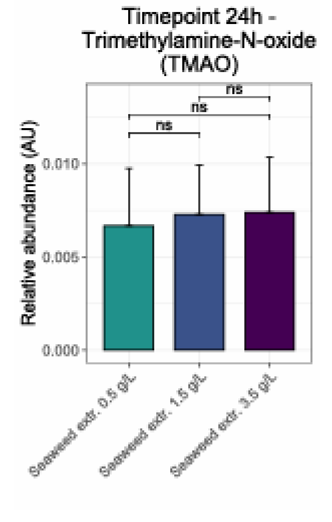 Timepoint 24h - Trimethylamine-N-oxide (TMAO)<br>Relative abundance (AU)<br>Seaweed extr. 0.5 g/L<br>Seaweed extr. 1.5 g/L<br>Seaweed extr. 3.5 g/L	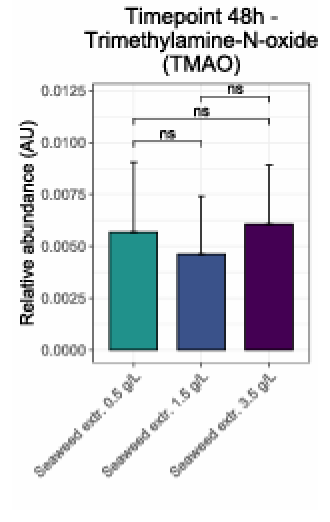 Timepoint 48h - Trimethylamine-N-oxide (TMAO)<br>Relative abundance (AU)<br>Seaweed extr. 0.5 g/L<br>Seaweed extr. 1.5 g/L<br>Seaweed extr. 3.5 g/L
2.	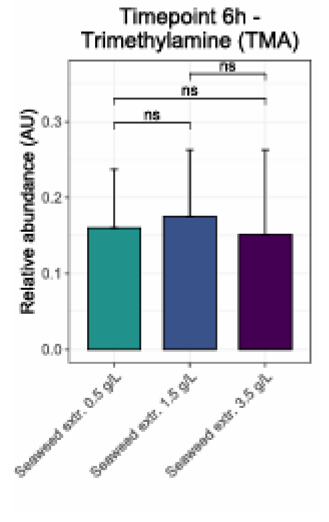 Timepoint 6h - Trimethylamine (TMA)<br>Relative abundance (AU)<br>Seaweed extr. 0.5 g/L<br>Seaweed extr. 1.5 g/L<br>Seaweed extr. 3.5 g/L	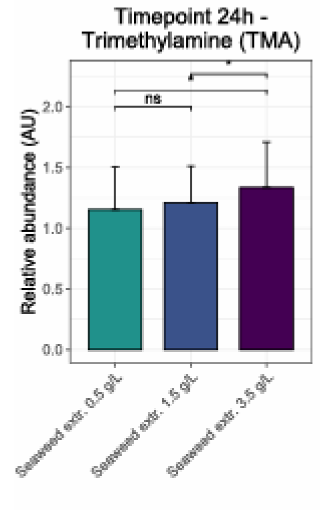 Timepoint 24h - Trimethylamine (TMA)<br>Relative abundance (AU)<br>Seaweed extr. 0.5 g/L<br>Seaweed extr. 1.5 g/L<br>Seaweed extr. 3.5 g/L	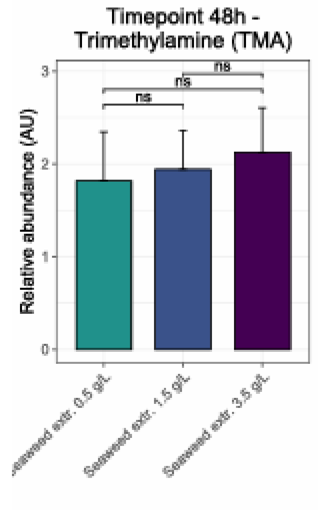 Timepoint 48h - Trimethylamine (TMA)<br>Relative abundance (AU)<br>Seaweed extr. 1.5 g/L<br>Seaweed extr. 3.5 g/L

3.

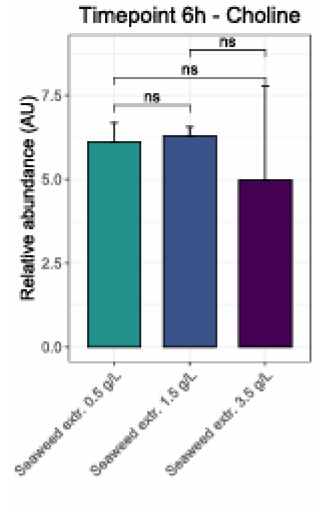
Timepoint 6h - Choline
Relative abundance (AU)
0.0
2.5
5.0
7.5
ns
ns
ns
Seaweed extr. 0.5 g/L
Seaweed extr. 1.5 g/L
Seaweed extr. 3.5 g/L

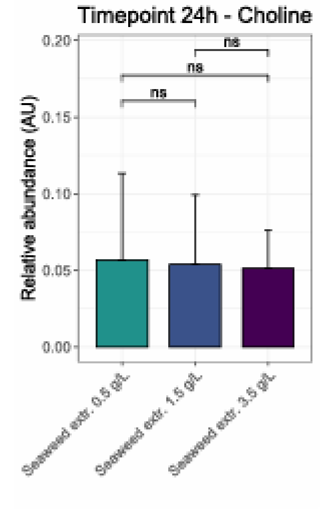
Timepoint 24h - Choline
Relative abundance (AU)
0.00
0.05
0.10
0.15
0.20
ns
ns
ns
Seaweed extr. 0.5 g/L
Seaweed extr. 1.5 g/L
Seaweed extr. 3.5 g/L

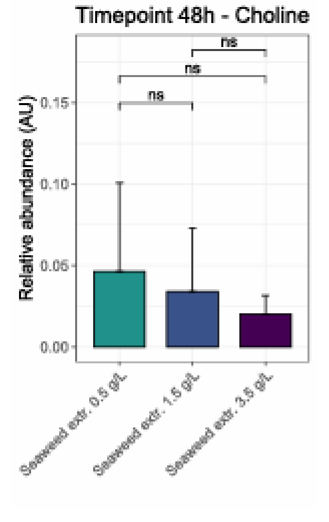
Timepoint 48h - Choline
Relative abundance (AU)
0.00
0.05
0.10
0.15
ns
ns
ns
Seaweed extr. 0.5 g/L
Seaweed extr. 1.5 g/L
Seaweed extr. 3.5 g/L

4.

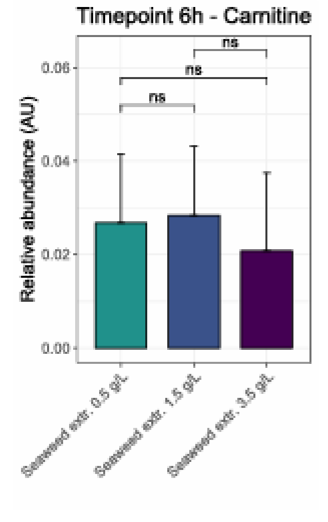
Timepoint 6h - Carnitine
Relative abundance (AU)
0.00
0.02
0.04
0.06
ns
ns
ns
Seaweed extr. 0.5 g/L
Seaweed extr. 1.5 g/L
Seaweed extr. 3.5 g/L

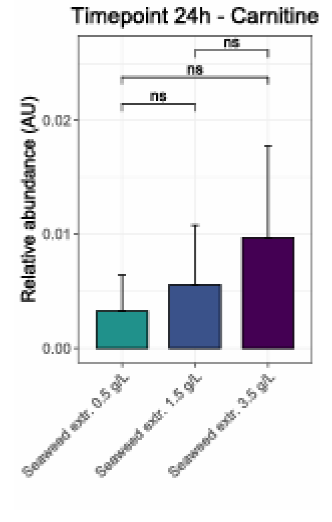
Timepoint 24h - Carnitine
Relative abundance (AU)
0.00
0.01
0.02
ns
ns
ns
Seaweed extr. 0.5 g/L
Seaweed extr. 1.5 g/L
Seaweed extr. 3.5 g/L

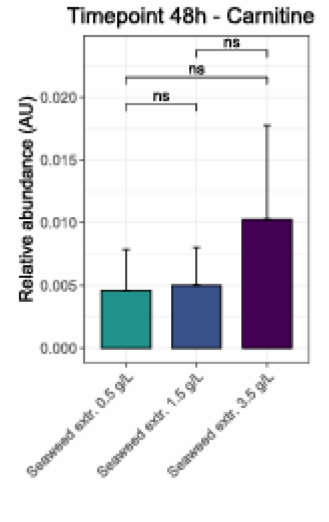
Timepoint 48h - Carnitine
Relative abundance (AU)
0.000
0.005
0.010
0.015
0.020
ns
ns
ns
Seaweed extr. 0.5 g/L
Seaweed extr. 1.5 g/L
Seaweed extr. 3.5 g/L

5.

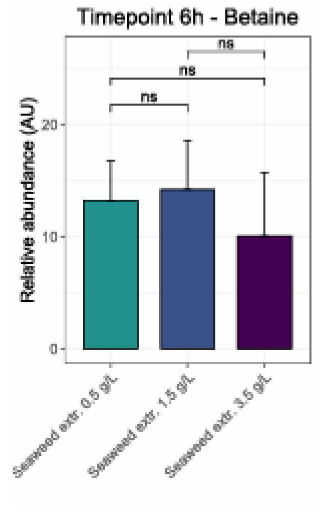
Timepoint 6h - Betaine
Relative abundance (AU)
0
10
20
ns
ns
ns
Seaweed extr. 0.5 g/L
Seaweed extr. 1.5 g/L
Seaweed extr. 3.5 g/L

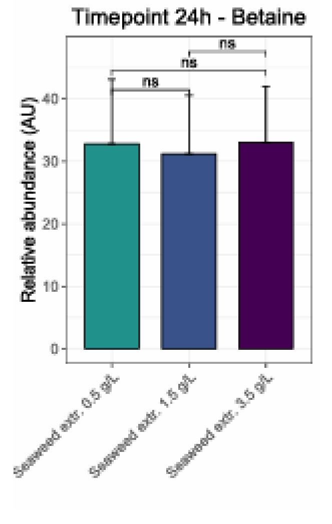
Timepoint 24h - Betaine
Relative abundance (AU)
0
10
20
30
40
ns
ns
ns
Seaweed extr. 0.5 g/L
Seaweed extr. 1.5 g/L
Seaweed extr. 3.5 g/L

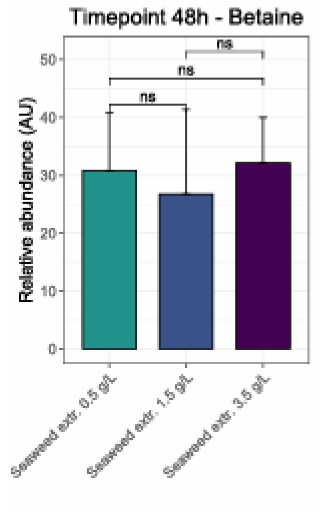
Timepoint 48h - Betaine
Relative abundance (AU)
0
10
20
30
40
50
ns
ns
ns
Seaweed extr. 0.5 g/L
Seaweed extr. 1.5 g/L
Seaweed extr. 3.5 g/L

6.

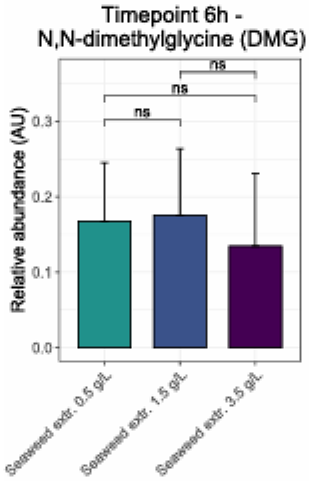
Timepoint 6h -
N,N-dimethylglycine (DMG)
Relative abundance (AU)
ns
ns
ns
0.0
0.1
0.2
0.3
Seaweed extr. 0.5 g/L
Seaweed extr. 1.5 g/L
Seaweed extr. 3.5 g/L

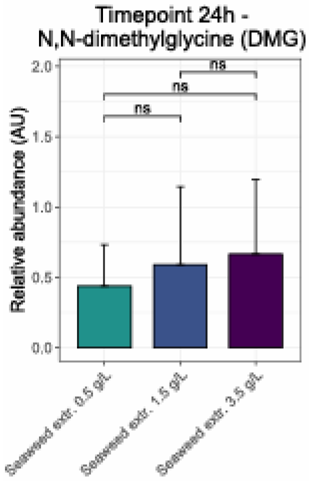
Timepoint 24h -
N,N-dimethylglycine (DMG)
Relative abundance (AU)
ns
ns
ns
0.0
0.5
1.0
1.5
2.0
Seaweed extr. 0.5 g/L
Seaweed extr. 1.5 g/L
Seaweed extr. 3.5 g/L

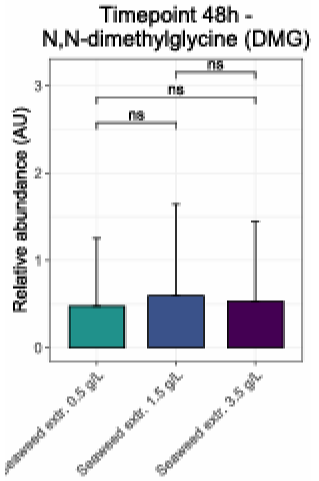
Timepoint 48h -
N,N-dimethylglycine (DMG)
Relative abundance (AU)
ns
ns
ns
0
1
2
3
Seaweed extr. 1.5 g/L
Seaweed extr. 3.5 g/L

7.

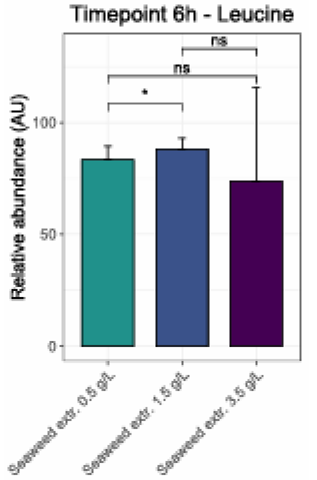
Timepoint 6h - Leucine
Relative abundance (AU)
ns
ns
*
0
50
100
Seaweed extr. 0.5 g/L
Seaweed extr. 1.5 g/L
Seaweed extr. 3.5 g/L

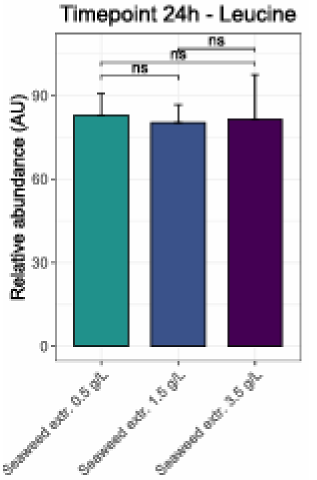
Timepoint 24h - Leucine
Relative abundance (AU)
ns
ns
ns
0
30
60
90
Seaweed extr. 0.5 g/L
Seaweed extr. 1.5 g/L
Seaweed extr. 3.5 g/L

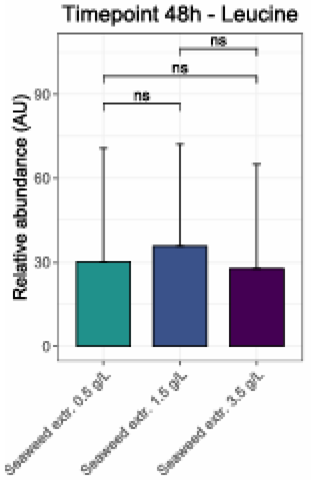
Timepoint 48h - Leucine
Relative abundance (AU)
ns
ns
ns
0
30
60
90
Seaweed extr. 0.5 g/L
Seaweed extr. 1.5 g/L
Seaweed extr. 3.5 g/L

8.

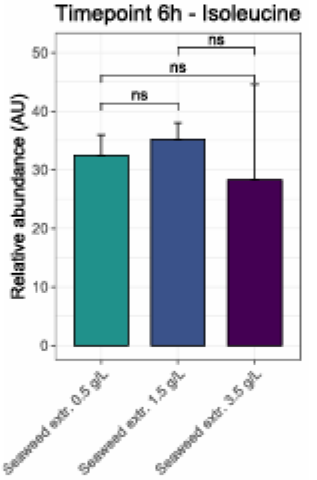
Timepoint 6h - Isoleucine
Relative abundance (AU)
ns
ns
ns
0
10
20
30
40
50
Seaweed extr. 0.5 g/L
Seaweed extr. 1.5 g/L
Seaweed extr. 3.5 g/L

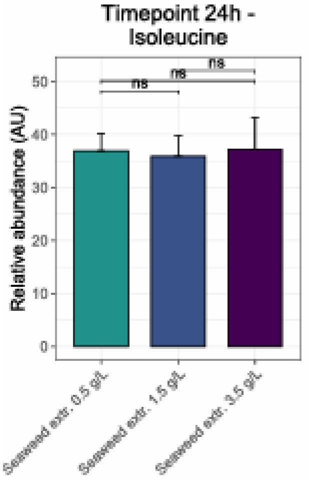
Timepoint 24h -
Isoleucine
Relative abundance (AU)
ns
ns
ns
0
10
20
30
40
50
Seaweed extr. 0.5 g/L
Seaweed extr. 1.5 g/L
Seaweed extr. 3.5 g/L

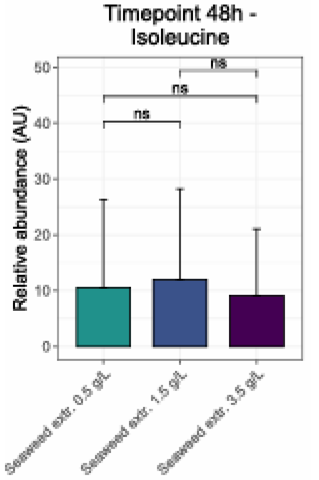
Timepoint 48h -
Isoleucine
Relative abundance (AU)
ns
ns
ns
0
10
20
30
40
50
Seaweed extr. 0.5 g/L
Seaweed extr. 1.5 g/L
Seaweed extr. 3.5 g/L

9.

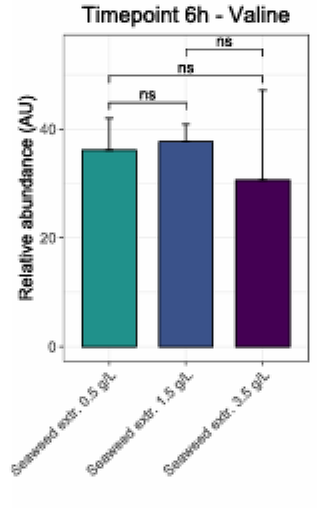
Timepoint 6h - Valine
Relative abundance (AU)
0
20
40
ns
ns
ns
Seaweed extr. 0.5 g/L
Seaweed extr. 1.5 g/L
Seaweed extr. 3.5 g/L

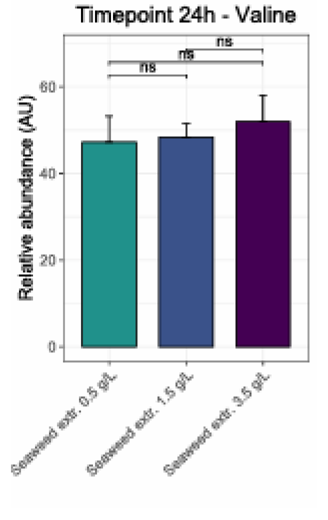
Timepoint 24h - Valine
Relative abundance (AU)
0
20
40
60
ns
ns
ns
Seaweed extr. 0.5 g/L
Seaweed extr. 1.5 g/L
Seaweed extr. 3.5 g/L

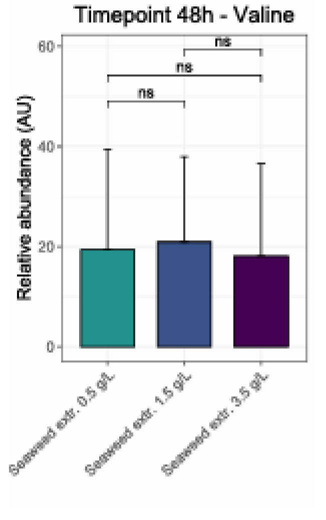
Timepoint 48h - Valine
Relative abundance (AU)
0
20
40
60
ns
ns
ns
Seaweed extr. 0.5 g/L
Seaweed extr. 1.5 g/L
Seaweed extr. 3.5 g/L

10.

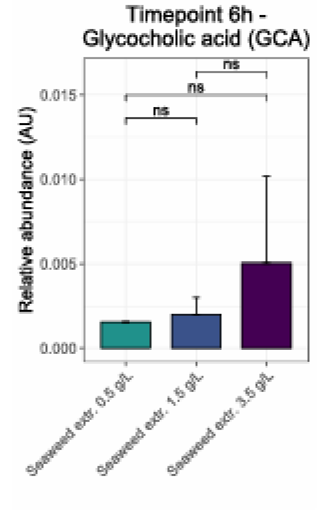
Timepoint 6h -
Glycocholic acid (GCA)
Relative abundance (AU)
0.000
0.005
0.010
0.015
ns
ns
ns
Seaweed extr. 0.5 g/L
Seaweed extr. 1.5 g/L
Seaweed extr. 3.5 g/L

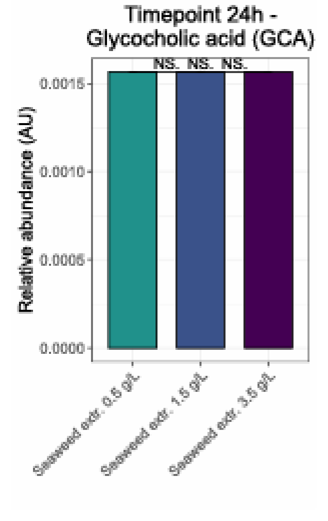
Timepoint 24h -
Glycocholic acid (GCA)
Relative abundance (AU)
0.0000
0.0005
0.0010
0.0015
NS. NS. NS.
Seaweed extr. 0.5 g/L
Seaweed extr. 1.5 g/L
Seaweed extr. 3.5 g/L

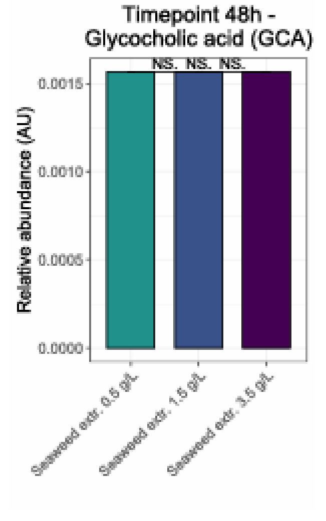
Timepoint 48h -
Glycocholic acid (GCA)
Relative abundance (AU)
0.0000
0.0005
0.0010
0.0015
NS. NS. NS.
Seaweed extr. 0.5 g/L
Seaweed extr. 1.5 g/L
Seaweed extr. 3.5 g/L

13.

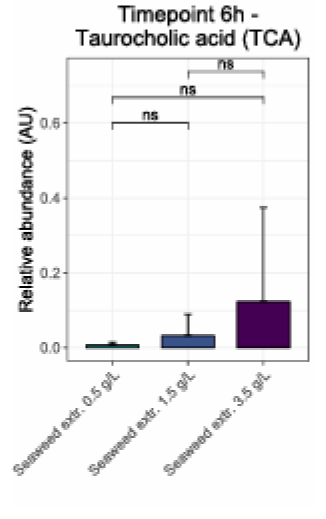
Timepoint 6h -
Taurocholic acid (TCA)
Relative abundance (AU)
0.0
0.2
0.4
0.6
ns
ns
ns
Seaweed extr. 0.5 g/L
Seaweed extr. 1.5 g/L
Seaweed extr. 3.5 g/L

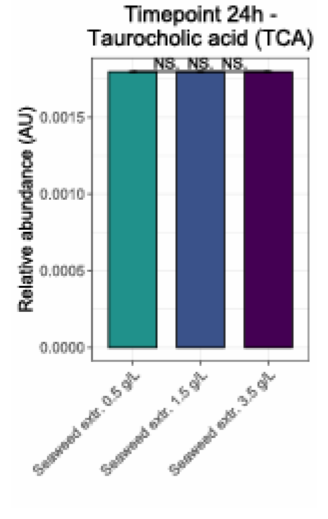
Timepoint 24h -
Taurocholic acid (TCA)
Relative abundance (AU)
0.0000
0.0005
0.0010
0.0015
NS. NS. NS.
Seaweed extr. 0.5 g/L
Seaweed extr. 1.5 g/L
Seaweed extr. 3.5 g/L

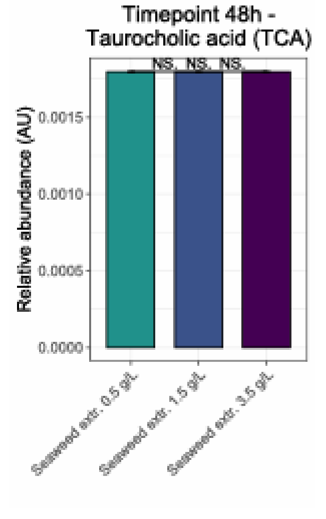
Timepoint 48h -
Taurocholic acid (TCA)
Relative abundance (AU)
0.0000
0.0005
0.0010
0.0015
NS. NS. NS.
Seaweed extr. 0.5 g/L
Seaweed extr. 1.5 g/L
Seaweed extr. 3.5 g/L

14.

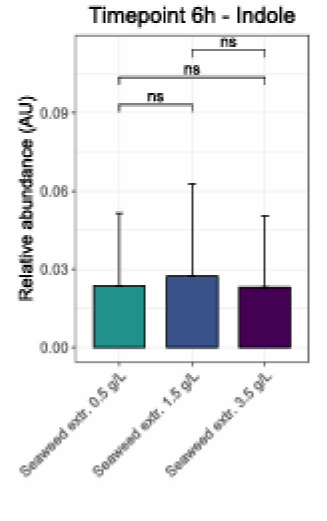

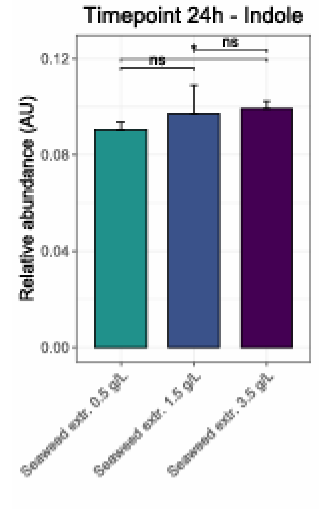

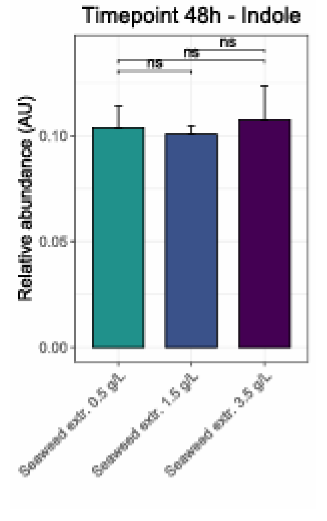

16.

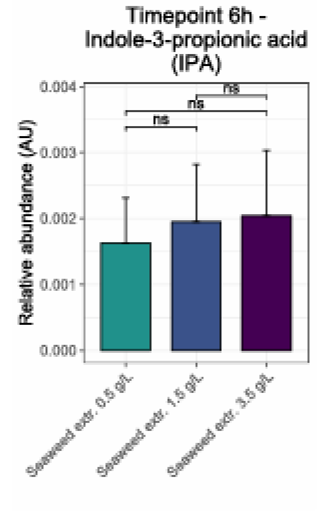

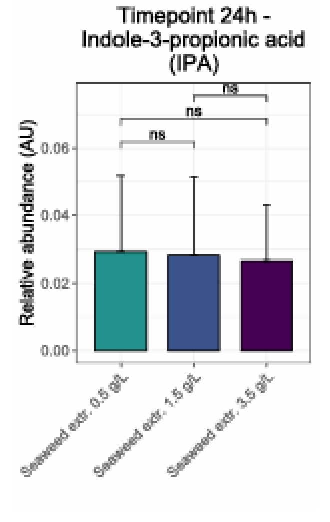

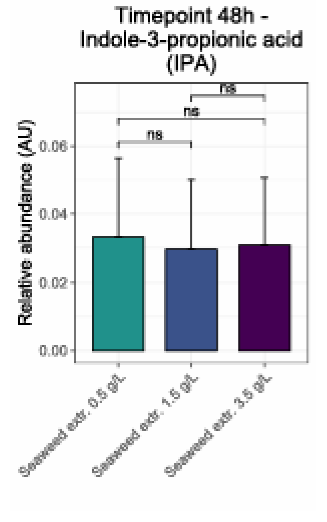

20.

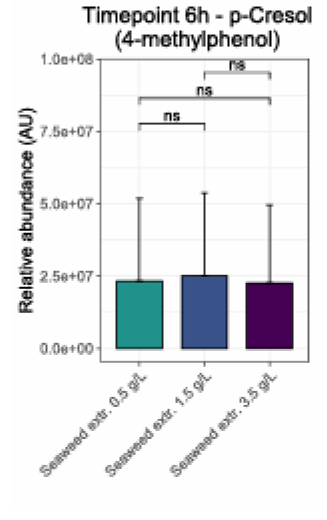

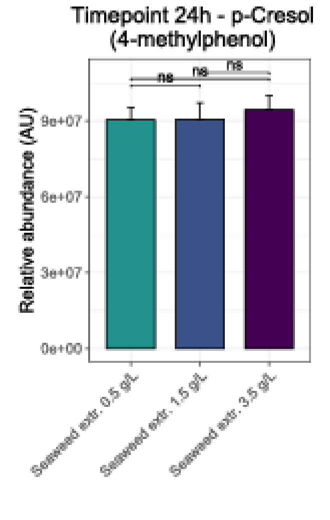

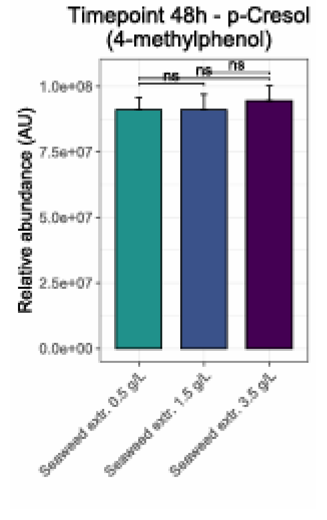

Figure S12. Bar plots of relative abundances at different timepoints (left, 6h timepoint; center 24h timepoint; right, 48h timepoint) of treatment conditions: seaweed extract 0.5 g/L, seaweed extract 1.5 g/L and seaweed extract 3.5 g/L. Averages are depicted over 5 replicates, as well as the standard deviation. Raw p-values from paired t-test results of comparison between

seaweed extracts are shown, with ns raw p-value > 0.05; * raw p-value ≤ 0.05; ** raw p-value ≤ 0.01; *** raw p-value ≤ 0.001.

Table S9 . p-values of the paired t-tests comparing metabolite levels between the different seaweed extracts at timepoint 6h. p-values depicted in bold show significant differences. Significance cutoffs are set at ns raw p-value > 0.05; * raw p value ≤ 0.05.

	Seaweed extracts comparison between	0.5 g/L vs 1.5 g/L		0.5 g/L vs 3.5 g/L		1.5 g/L vs 3.5 g/L	
nr	metabolite	*p*-val.	Signif.	*p*-val.	Signif.	*p*-val.	Signif.
1	Trimethylamine-N-oxide (TMAO)	0.441	ns	0.599	ns	0.166	ns
2	Trimethylamine (TMA)	0.112	ns	0.722	ns	0.273	ns
3	Choline	0.615	ns	0.441	ns	0.367	ns
4	Carnitine	0.714	ns	0.225	ns	0.069	ns
5	Betaine	0.200	ns	0.401	ns	0.353	ns
6	N,N-dimethylglycine (DMG)	0.310	ns	0.494	ns	0.431	ns
7	Leucine	**0.030**	*	0.666	ns	0.508	ns
8	Isoleucine	0.187	ns	0.583	ns	0.436	ns
9	Valine	0.343	ns	0.563	ns	0.432	ns
10	Glycocholic acid (GCA)	0.374	ns	0.206	ns	0.192	ns
13	Taurocholic acid (TCA)	0.321	ns	0.345	ns	0.352	ns
14	Indole	0.362	ns	0.901	ns	0.444	ns
16	Indole-3-propionic acid (IPA)	0.291	ns	0.348	ns	0.519	ns
20	p-Cresol (4-methylphenol)	0.108	ns	0.844	ns	0.404	ns

Table S10 . p-values of the paired t-tests comparing metabolite levels between the different seaweed extracts at timepoint 24h. p-values depicted in bold show significant differences. Significance cutoffs are set at ns raw p-value > 0.05; * raw p value ≤ 0.05.

	Seaweed extracts comparison between	0.5 g/L vs 1.5 g/L		0.5 g/L vs 3.5 g/L		1.5 g/L vs 3.5 g/L	
nr	metabolite	*p*-val.	Signif.	*p*-val.	Signif.	*p*-val.	Signif.
1	Trimethylamine-N-oxide (TMAO)	0.629	ns	0.431	ns	0.925	ns
2	Trimethylamine (TMA)	0.233	ns	**0.022**	*	**0.035**	*
3	Choline	0.780	ns	0.803	ns	0.871	ns
4	Carnitine	0.163	ns	0.162	ns	0.321	ns
5	Betaine	0.297	ns	0.892	ns	0.395	ns
6	N,N-dimethylglycine (DMG)	0.271	ns	0.136	ns	0.365	ns
7	Leucine	0.499	ns	0.806	ns	0.817	ns
8	Isoleucine	0.703	ns	0.888	ns	0.590	ns
9	Valine	0.462	ns	0.100	ns	0.177	ns
10	Glycocholic acid (GCA)	NA	ns	NA	ns	NA	ns
13	Taurocholic acid (TCA)	NA	ns	NA	ns	NA	ns
14	Indole	0.217	ns	**0.013**	*	0.662	ns
16	Indole-3-propionic acid (IPA)	0.736	ns	0.661	ns	0.763	ns
20	p-Cresol (4-methylphenol)	0.966	ns	0.131	ns	0.432	ns

Table S11. p-values of the paired t-tests comparing metabolite levels between the different seaweed extracts at timepoint 48h. p-values depicted in bold show significant differences. Significance cutoffs are set at ns raw p-value > 0.05.

	Seaweed extracts comparison between	0.5 g/L vs 1.5 g/L		0.5 g/L vs 3.5 g/L		1.5 g/L vs 3.5 g/L	
nr	metabolite	*p*-val.	Signif.	*p*-val.	Signif.	*p*-val.	Signif.
1	Trimethylamine-N-oxide (TMAO)	0.096	ns	0.765	ns	0.192	ns
2	Trimethylamine (TMA)	0.071	ns	0.140	ns	0.271	ns
3	Choline	0.265	ns	0.258	ns	0.333	ns
4	Carnitine	0.340	ns	0.124	ns	0.185	ns
5	Betaine	0.220	ns	0.475	ns	0.182	ns
6	N,N-dimethylglycine (DMG)	0.368	ns	0.508	ns	0.401	ns
7	Leucine	0.492	ns	0.919	ns	0.709	ns
8	Isoleucine	0.150	ns	0.880	ns	0.772	ns
9	Valine	0.669	ns	0.915	ns	0.789	ns
10	Glycocholic acid (GCA)	NA	ns	NA	ns	NA	ns
13	Taurocholic acid (TCA)	NA	ns	NA	ns	NA	ns
14	Indole	0.555	ns	0.544	ns	0.444	ns
16	Indole-3-propionic acid (IPA)	0.106	ns	0.518	ns	0.601	ns
20	p-Cresol (4-methylphenol)	0.940	ns	0.107	ns	0.180	ns

Changes in microbial community composition

Two techniques were combined according to Vandeputte et al. (2017) [20] to map the community shifts induced by the different treatments in large detail:

- **16S rRNA-targeted Illumina sequencing**, providing proportional abundances of different taxa at different phylogenetic levels (microbial phylum, family and genus level).
- Accurate quantification of total bacterial cells in the samples through **flow cytometry**.

Combining the phylogenetic information obtained with 16S Illumina sequencing with the accurate enumeration of the cell counts via flow cytometry yields insight in quantitative abundances of the different taxonomic entities inside the reactors.

Community composition in the original stool samples

Microbial community composition at the start in the blank conditions for donors A, B, C, D and E is representative for community composition in the original fecal inocula. Community composition is visualized for each individual donor using jitter plots, and is reported on the phylum, family, genus, and OTU level (Figure S13). Per level, the top 20 most abundant taxa are displayed.

The donors' colonic microbiota primarily consisted of members of the *Firmicutes*, *Bacteroidetes,* and *Actinobacteria* phyla. Within the *Firmicutes* phylum, *Lachnospiraceae* and *Ruminococcaceae* were the dominant families across donors. *Bacteroidetes* were primarily represented by *Bacteroidaceae* and/or *Prevotellaceae* in each donor, except for donor C, where *Rikenellaceae* was dominant. The *Actinobacteria* phylum was primarily represented by *Bifidobacteriaceae* in donors C and D, and by *Coriobacteriaceae* in donor A. The other donors were characterized by lower *Actinobacteria* levels.

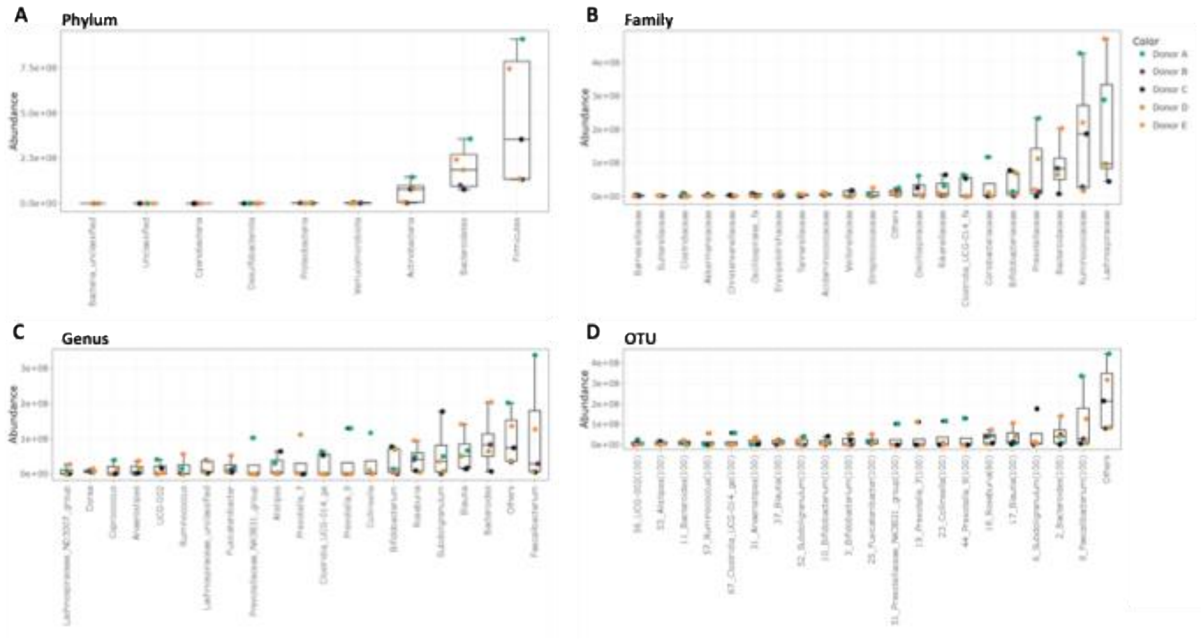

Figure S13: Jitter plots showing absolute abundances (cells/mL) of the top 20 most abundant phyla (A), families (B), genera (C) and OTUs (corresponding genus classifications indicated as 'OTU number_genus') (D) in the blank at the start of the incubation, thus representing the donors' original fecal inocula. Each color represents a donor (donor A-B-C-D-E).

Community composition after treatment

An assessment was made of the impact of the various treatments on the microbial communities of donors A, B, C, D and E 48h after start of incubation. In order to do so, alpha- and beta-diversity were calculated, and differential abundance analysis was performed with LEfSe and treeclimbR.

To examine the effects of the different treatments on bacterial diversity within conditions, ***alpha*-diversity** was calculated. ***Beta*-diversity,** or differences in community composition between conditions, was assessed and presented in a hierarchical clustering dendrogram and in a scatter plot (Discriminant Analysis of Principle Components).

LEfSe and treeclimbR were used to identify which bacterial groups were significantly altered by the different treatments across the donors. LEfSe and treeclimbR identify features, in this case taxa, which characterize differences between two biological conditions. By looking at consistencies across donors (in this analysis, each donor is considered a 'replicate' measurement), the effect becomes independent of interindividual variation.

Effects on alpha-diversity

The impact of treatment on alpha-diversity is shown in Figure S14. Observed and Chao1 indices indicate that seaweed extract increased **species richness** as compared to inulin and the untreated control. A positive dose-response correlation was observed. In contrast, inulin treatment was associated with lower alpha-diversity as compared to the untreated control. Drops in species richness upon treatment can be attributed to high affinity of a select number of

bacteria for the provided substrate, resulting in their outgrowth. These results suggest that a broader range of bacteria was enriched by Oceanium's seaweed extract as compared to inulin.

In contrast, **species evenness** (represented by the Shannon and Simpson indices) decreased upon treatment with the seaweed extract, probably attributed to the fact that, amongst the broader range of product fermenters, the more efficient product fermenters were able to proliferate and became more abundant as compared to less efficient fermenters. The strongest impact on evenness was observed with the 1.5 g/L dose, meaning that in this case a dose-response was not observed.

Effects on beta-diversity

The effect of treatment on beta-diversity, and therefore the extent of the impact on microbial community composition, is shown in Figure S15. Treatment with the seaweed extract or inulin had a significant impact on community composition, as indicated by the segregation from the blank in both beta-diversity plots. Communities treated with the highest seaweed concentrations (1.5 and 3.5 g/L) shared more similarities in terms of community composition as compared to the lowest concentration (0.5 g/L). Community composition was distinct from the fiber control.

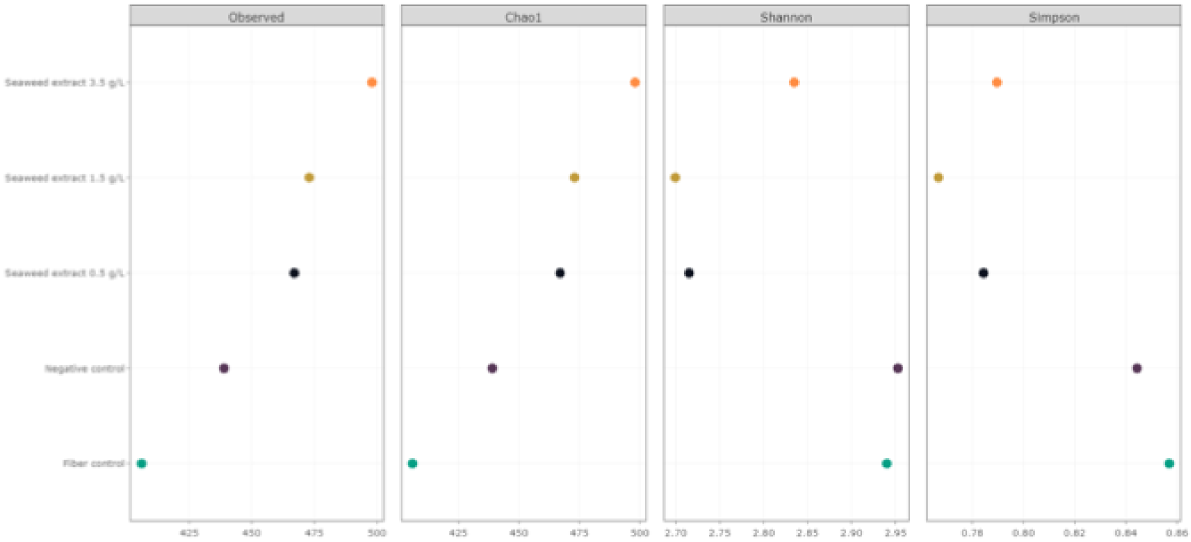

Figure S14: *Alpha* diversity in the various conditions 48h after start of incubation (average across the donors). The effect of three concentrations of a seaweed extract (0.5, 1.5 and 3.5 g/L) on microbial community composition was assessed and compared to an untreated control and a fiber control.

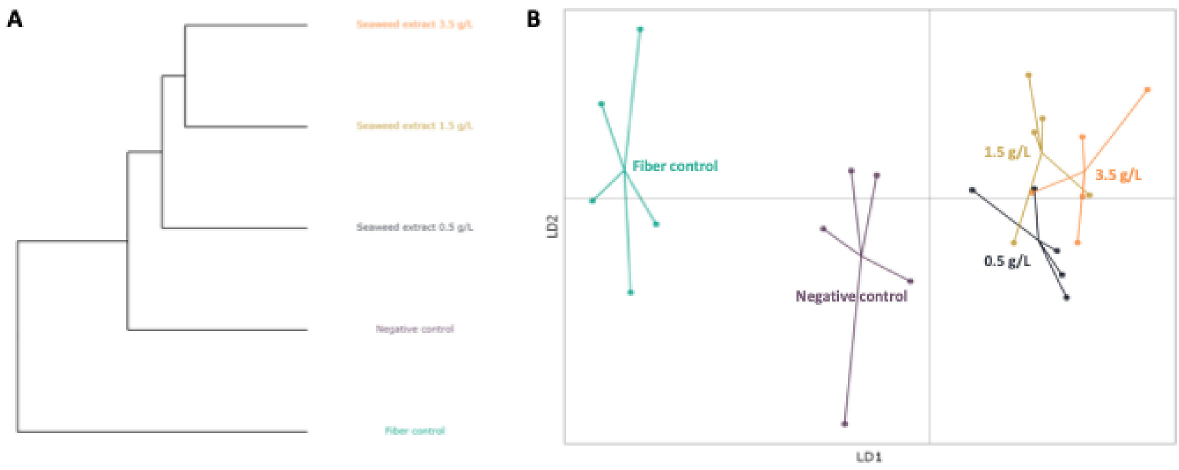

Figure S15: Beta diversity presented by hierarchical clustering (A) and Discriminant analysis of principal components (DAPC) (B) to quantify the dissimilarity in community composition amongst the various conditions 48h after start of the incubation. The impact of three concentrations of a seaweed extract (0.5, 1.5 and 3.5 g/L) on gut microbial community composition of five healthy donors was studied. A fiber control (3.5 g/L inulin) and a negative control (blank) were included as reference conditions. Each color represents a different condition (treatment or control) and each dot in the DAPC plot represents one of the five donors. LD1 and LD2 are Linear Discriminants.

Effects on taxa abundances

LEfSe (genus level) and treeclimbR (genus level and higher) analyses were performed to detect which bacteria were significantly affected by treatment (across donors), using the negative control as a reference (Figure S18-Figure S21) for the seaweed extract and Figure S24-Figure S25 for the fiber control). LEfSe detected no statistically significant differences upon treatment with 0.5 g/L or 1.5 g/L seaweed extract, but biologically significant differences were detected using treeclimbR. Abundances in the various conditions of genera and families that were affected by treatment with the seaweed extract are shown in Figure S16-Figure S17, and of those affected by inulin treatment in Figure S22-Figure S23.

Treatment with 3.5 g/L, 1.5 g/L or 0.5 g/L of the **seaweed extract** was associated with compositional shifts within the *Firmicutes* phylum, attributed to uncultured/unclassified *Oscillospiraceae*, *Butyricicoccus* spp., *Veillonellaceae* and/or *Lachnospiraceae* (related to *Eubacterium* spp.). TreeclimbR analysis indicated that the increased abundance of uncultured *Oscillospiraceae* species was biologically significant with the highest dose of seaweed extract (**3.5 g/L**), but that statistical significance was not reached (Figure S20). In addition, LEfSe indicated that treatment with the highest dose significantly increased the abundance of the *Butyricicoccus* spp. (LDA=1.3) and *Lachnospiraceae* spp. (related to *Eubacterium)* (LDA=1.9) (Figure S21), but the threshold of biological significance (LDA=2) was not reached, suggesting a mild enrichment effect. TreeclimbR indicated that unclassified *Oscillospiraceae* spp. and the *Veillonellaceae* family, represented by genera *Veillonella*, *Dialister* and *Megasphaera,* were increased in abundance upon treatment with the lowest dose of the seaweed extract (**0.5 g/L**), but significance across the donors was not reached in these cases (Figure S23).

Several bacteria were more abundant in blank as compared to the seaweed extract, suggesting inhibition by treatment. However, as the analysis is based on relative data, this can also mean that abundances of these bacteria were not altered by treatment (relative abundances going down due to the enrichment of several other community members, falsely suggesting inhibition). Such community shifts involved members of the *Firmicutes* phylum, more specifically *Eisenbergiella*, *Enterococcus*, and *Lactiplantibacillus* for treatment with the medium (**1.5 g/L**) and high doses (**3.5 g/L**) (Figure S18 and Figure S20), and *Alistipes* (*Bacteroidetes*) and *Akkermansia* (*Verrucomicrobiota*) for treatment with the high dose (Figure S19). Regardless of the above, these results indicate that these organisms were not enriched by treatment.

Inulin treatment (3.5 g/L) was associated with the biologically and statistically significant enrichment of *Blautia* and unclassified *Lachnospiraceae* (Figure S24). In addition, several taxa were enriched for which the threshold of biological significance was reached, but which were not statistically supported (due to interindividual variation). These included unclassified *Enterobacteriaceae*, *Faecalibacterium* and *Veillonellaceae*. TreeclimbR analysis also indicated several bacteria with lower abundances in inulin condition as compared to blank, including *Parabacteroides*, *Flavonifractor*, *Oscillospiraceae* UCG-002, *Akkermansia* and *Marvinifilaceae*. In addition, LEfSe analysis indicated a significantly lower abundance of *Coprococcus* upon fiber treatment (Figure S25).

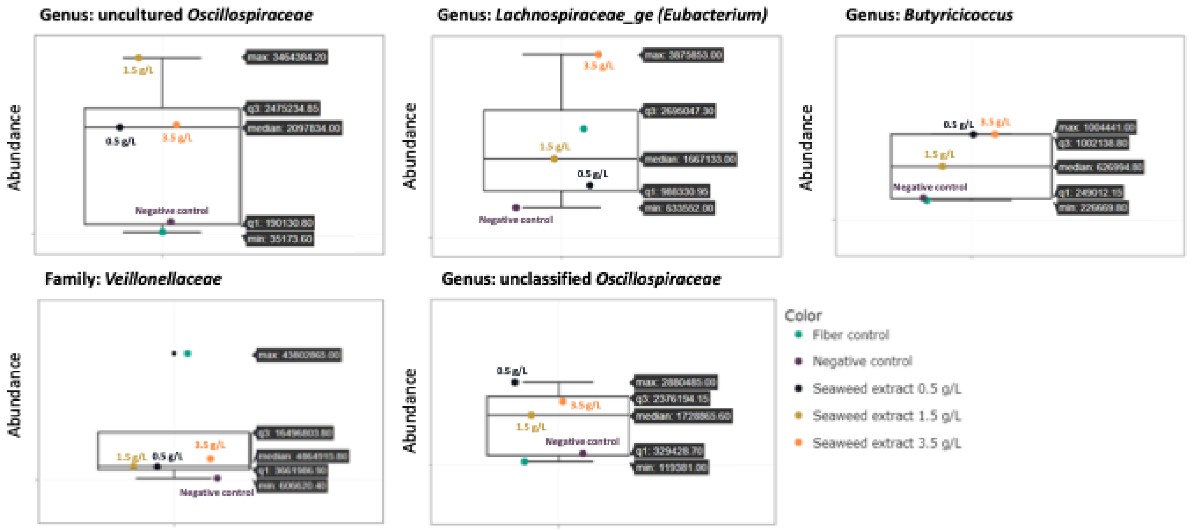

Figure S16: Jitter plots showing average absolute abundances (cells/mL) of bacterial genera and families which were enriched upon treatment with the seaweed extract (0.5, 1.5 and/or 3.5 g/L) as compared to the negative control 48h after the start of the incubation, as indicated by LEfSe and/or treeclimbR. Each dot represents the average across five donors. Each color represents a different condition (treatment or control).

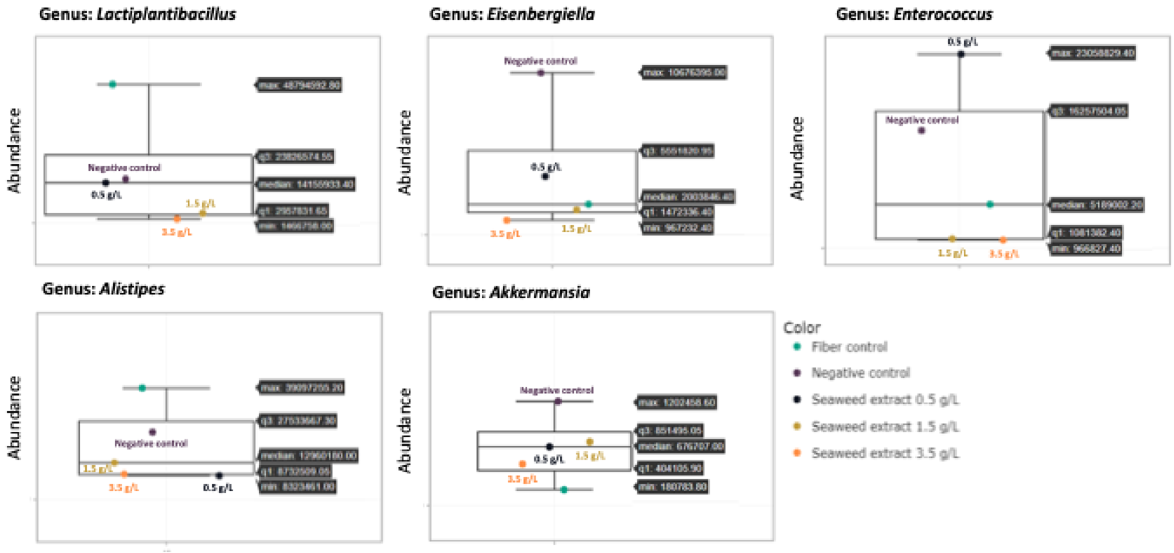

Figure S17: Jitter plots showing average absolute abundances (cells/mL) of bacterial genera and families with lower abundances in treatment (0.5 g/L, 1.5 g/L or 3.5 g/L seaweed extract) than blank as compared to the negative control 48h after the start of the incubation, as indicated by LEfSe and/or treeclimbR. Each dot represents the average across five donors. Each color represents a different condition (treatment or control).

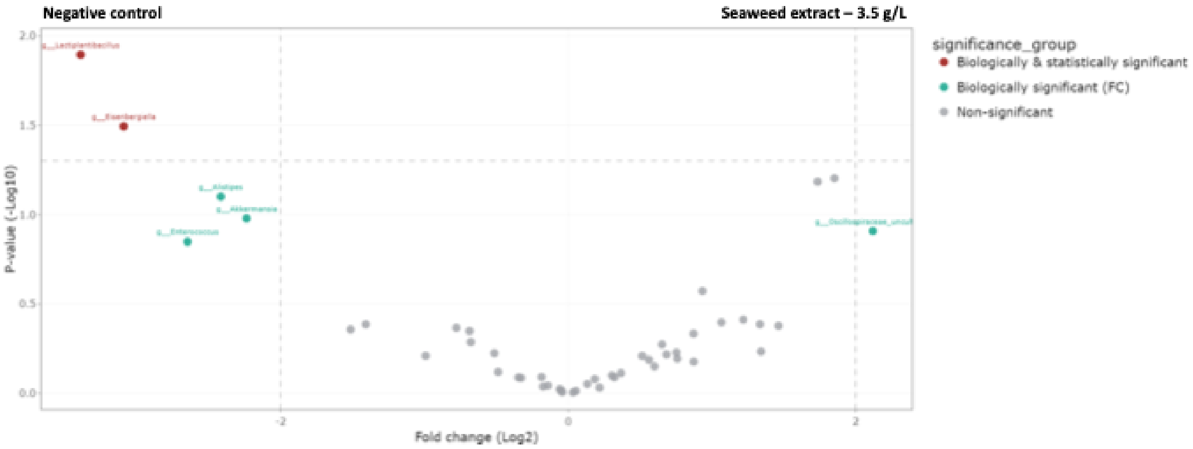

Figure S18: Differential abundance analysis (treeclimbR) to identify differences in community composition at various taxonomic levels between 3.5 g/L seaweed extract (right) and negative control (left) 48h after the start of incubation. The obtained scatter plot classifies taxa into four categories based on abundances in compared conditions: a) not significant and not biologically relevant (grey), b) biologically relevant, but not statistically significant (green), c) statistically significant, but not biologically relevant (blue), and d) biologically and statistically significant (red).

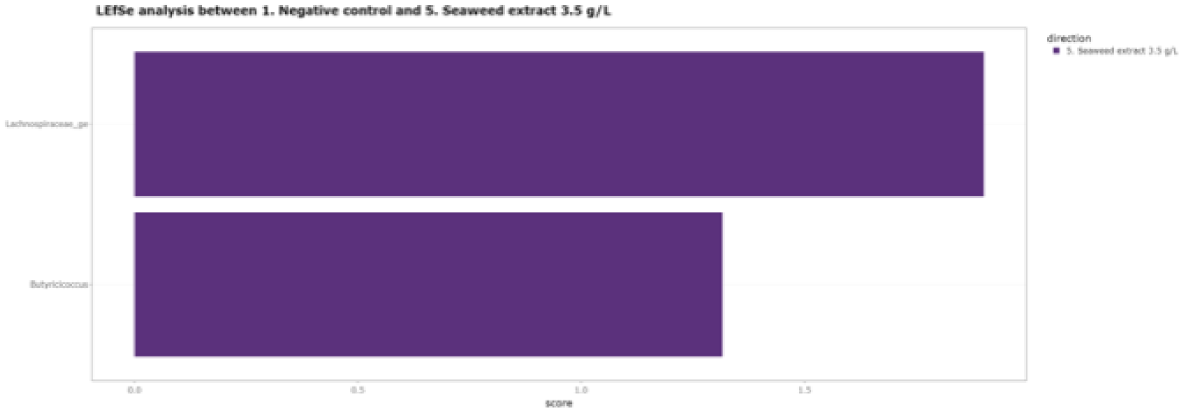

Figure S19: LEfSe bar plot showing significantly altered bacterial genera between control and condition with 3.5 g/L seaweed extract. The sections highlighted in purple represent features that are more abundant in the seaweed extract compared to the negative control. The x-axis represents the LDA score (measure of effect size).

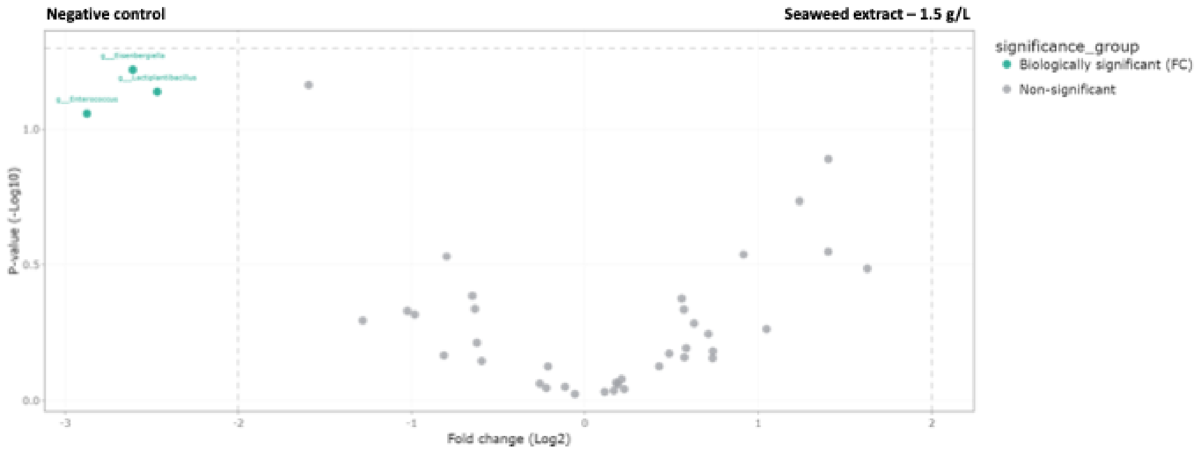

Figure S20: Differential abundance analysis (treeclimbR) to identify differences in community composition at various taxonomic levels between 1.5 g/L seaweed extract (right) and negative control (left) 48h after the start of incubation. The obtained scatter plot classifies taxa into four categories based on abundances in compared conditions: a) not significant and not biologically relevant (grey), b) biologically relevant, but not statistically significant (green), c) statistically significant, but not biologically relevant (blue), and d) biologically and statistically significant (red).

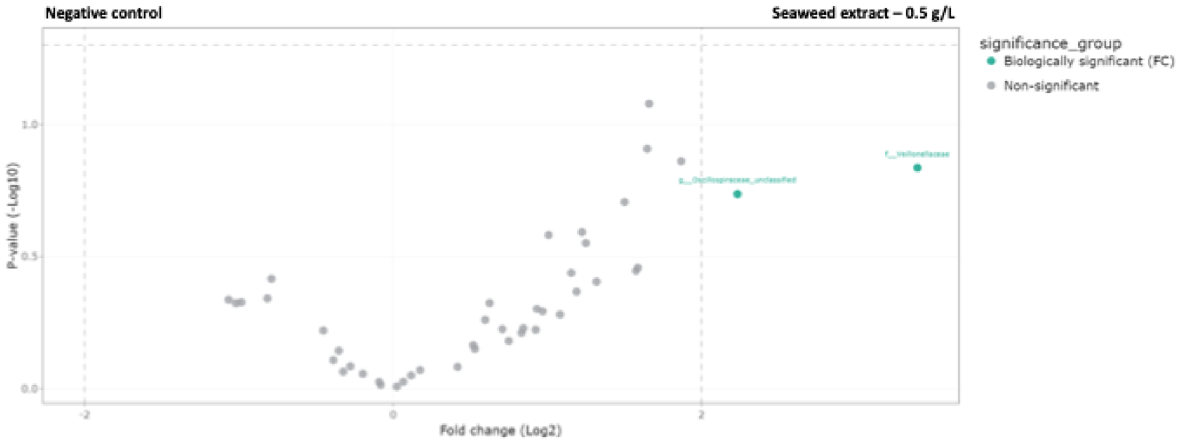

Figure S21: Differential abundance analysis (treeclimbR) to identify differences in community composition at various taxonomic levels between 0.5 g/L seaweed extract (right) and negative control (left) 48h after the start of incubation. The obtained scatter plot classifies taxa into four categories based on abundances in compared conditions: a) not significant and not biologically relevant (grey), b) biologically relevant, but not statistically significant (green), c) statistically significant, but not biologically relevant (blue), and d) biologically and statistically significant (red).

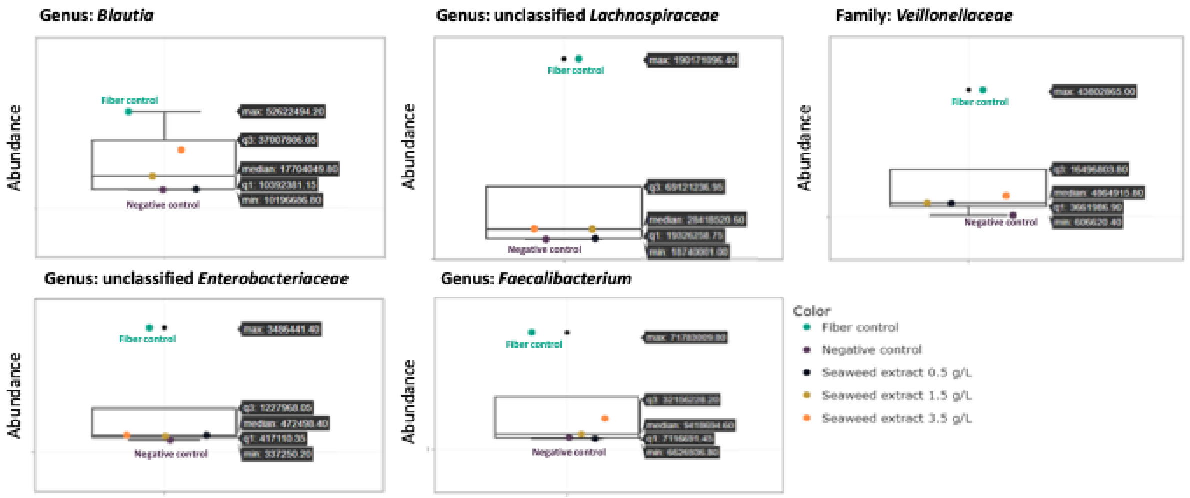

Figure S22: Jitter plots showing average absolute abundances (cells/mL) of bacterial genera and families which were enriched upon treatment with fiber control as compared to the negative control 48h after the start of the incubation, as indicated by LEfSe and/or treeclimbR. Each dot represents the average across five donors. Each color represents a different condition (treatment or control).

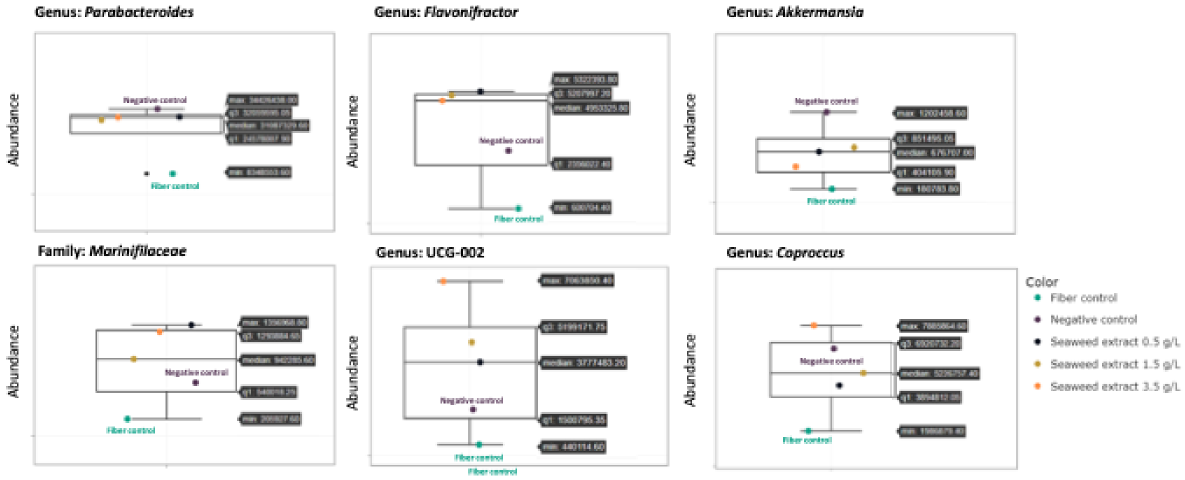

Figure S23: Jitter plots showing average absolute abundances (cells/mL) of bacterial genera and families with lower abundances in fiber control than blank 48h after the start of the incubation, as indicated by LEfSe and/or treeclimbR. Each dot represents the average across five donors. Each color represents a different condition (treatment or control).

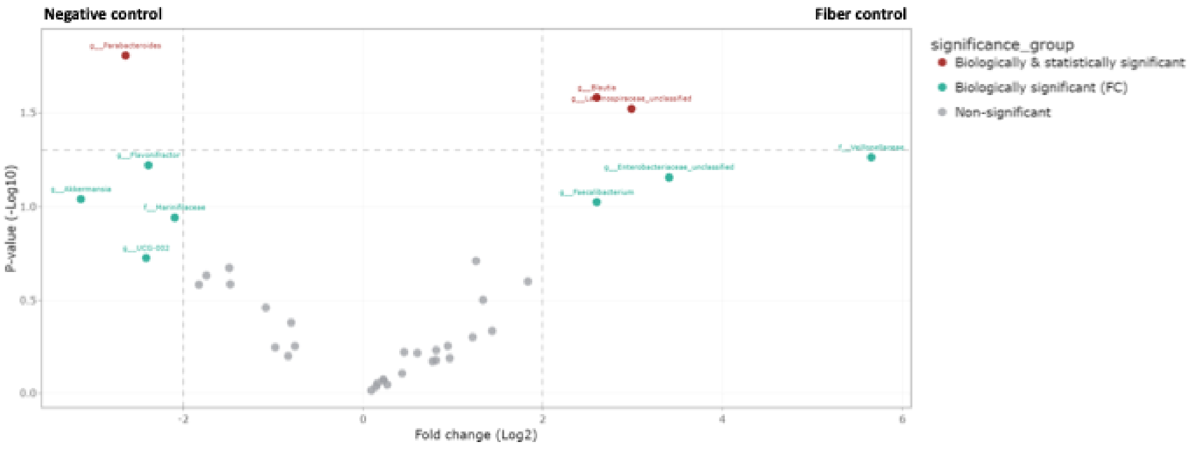

Figure S24: Differential abundance analysis (treeclimbR) to identify differences in community composition at various taxonomic levels between fiber control (right) and negative control (left) 48h after the start of incubation. The obtained scatter plot classifies taxa into four categories based on abundances in compared conditions: a) not significant and not biologically relevant (grey), b) biologically relevant, but not statistically significant (green), c) statistically significant, but not biologically relevant (blue), and d) biologically and statistically significant (red).

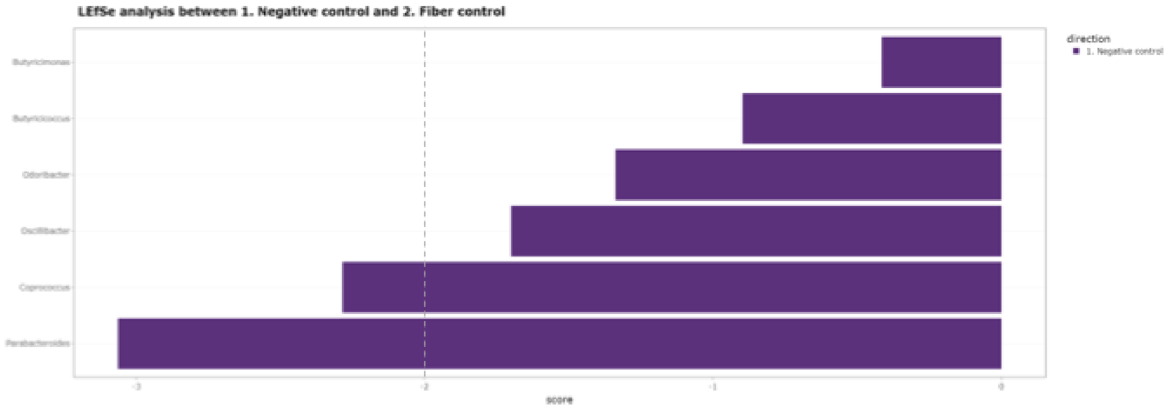

Figure S25: LEfSe bar plot showing significantly altered bacterial genera between control and condition with fiber control. The sections highlighted in purple represent features that more abundant in the negative control as compared to the fiber control. The x-axis represents the LDA score (measure of effect size), with LDA scores of +2 (generally accepted asbiologically relevant) indicated by dotted lines.

Conclusions

The aim of the project was to assess the impact of one of Oceanium's seaweed extracts on metabolites produced by the microbiome upon fermentation, and to investigate which microbial shifts were responsible for the observed findings. To this end, the impact of three concentrations of the extracton the microbiome of five healthy adult donors was investigated and compared to a negative control and a fiber control (inulin Frutafit TEX), using ProDigest's short-term single stage colonic simulation.

Overall, **pH** profiles indicated that the fermentation processes in the colonic simulations proceededunder conditions optimal to support growth of a wide diversity of gut microbial community member (pH varied between 5.74-6.48), enabling cross-feeding interactions, if any. This provides a solid baseline to evaluate effects of the investigated products on the colonic microbiota.

The seaweed extract had mild stimulatory effects on **gas production** at highest concentration (3.5 g/L) and is not expected to cause discomfort in the host. This contrasts with the fiber control, which, when tested at the same concentration, resulted in high gas pressures in early phases of fermentation. Inulin is thus more likely to cause bloating and discomfort to the host. Gas production was not affected bythe seaweed extract when dosed at lower concentration (0.5 or 1.5 g/L).

Fermentation of the seaweed extract was associated with mild stimulation of primary metabolite **acetate** in most donors, with higher doses giving higher acetate yields. **Lactate** production was not affected by treatment. Inulin had a stronger acetogenic effect than the seaweed extract, and stimulated lactate production also. Stimulation of acetate and lactate production in response to inulin treatment was observed in all donors. Treatment with seaweed extract significantly stimulated **propionate and butyrate** production compared to the negative control, with higher doses giving higher SCFA yields. However, in this case also the stimulatory effects were less pronounced as compared to inulin. In the case of inulin significance was not reached due to greater inter-donor variation. The butyrogenic effect of inulin was not consistent across the donors, with two of five donors not responding to treatment. This is in contrast with the seaweed extract, where the butyrogenic effect was observed in each donor, yet with smaller effect size.

In the light of this study, it is important to consider **some key differences between Frutafit TEX inulin and the seaweed extract**. The inulin applied in this study was characterized by high purity, and therefore the pre-digestion step was not necessary. The full product was thus added to the reactors. In contrast, the seaweed extract was subjected to a pre-digestion step, and part of the protein fraction (11%) in the product was absorbed during simulation of small intestinal passage, meaning that a slightly lower amount of product finally entered the colon reactors. This contributed to the overall finding that lower levels of the various endpoints were obtained with the seaweed extract as compared to the inulin type applied in this study.

Metagenomic analysis confirmed that the seaweed extract was well fermented by the gut microbiota, as it induced an increase in bacterial biomass and species richness. The biomass increase was substantially lower as compared to inulin, likely attributed to lower substrate concentrations reaching the colon after pre-digestion. However, the product's effect on alpha-diversity was improved, as the seaweed extract resulted in significantly higher species richness. The seaweed extract resulted in the enrichment of uncultured/unclassified *Oscillospiraceae*,

Butyricicoccus, *Veillonellaceae* and/or *Lachnospiraceae* (related to *Eubacterium*). *Veillonellaceae*, represented by *Veillonella*, *Dialister* and *Megasphaera*, is a collection of mostly acetate and/or propionate producing bacterial species positively correlated with gut-health. This enrichment may explain the product's propionogenic effect. Likewise, the *Oscillospiraceae* family, a large and diverse SCFA-producing family, is generally associated with gut-health, and, together with the enrichment of the unknown *Lachnoclostridium* genus and *Butyricicoccus*, was likely responsible for the butyrogenic treatment effect. Biologically or statistically significant bacterial enrichments were not observed for the medium product dose, probably attributed to a high degree of interindividual variation. Like the seaweed extract, inulin enriched *Veillonellaceae*, leading to elevated propionate-production. In addition, several butyrate-producers known to positively affect gut-health were stimulated upon inulin treatment, including *Blautia*, *Faecalibacterium* and unclassified *Lachnospiraceae*, thus explaining inulin's butyrogenic effect.

Metabolomic analysis revealed that the production of trimethylamine-N-oxide (TMAO) was significantly increased upon inulin treatment but not by the seaweed extract, already at 24h of incubation. TMAO is a downstream product of the TMA metabolism, and is mechanistically linked to increased cardiovascular risk [143], serving also as a prognostic factor of cardiovascular mortality [144], thus pointing towards a negative health impact of inulin as compared to seaweed extract. In contrast, although not significant, TMA levels were lower in inulin control than blank, which may indicate higher conversion of TMA into TMAO. Production of carnitine, to which numerous beneficial effects have been attributed (including protection against obesity-induced insulin resistance and myocardial ischemia, as well as the reduction of hypertension and obesity [145,146], was increased in the seaweed extract samples as compared to inulin samples. Furthermore, concentrations of indole, and its derivative indole-3-propionic acid (IPA), reduced upon inulin treatment, but not upon treatment with the seaweed extract. Since indole and IPA are negatively associated with advanced atherosclerosis and metabolic disorders [147,S148], the lowered production of indole, as induced by inulin, is negative for cardiac health. Lastly, p-Cresol was decreased upon treatment with inulin and the seaweed extracts, but the decrease resulting from inulin treatment was significantly stronger as compared to seaweed extract. In chronic kidney disease (CKD), *p*-cresol accumulates in plasma where it increases oxidative stress and influences mitochondrial function. It is further linked to CKD-associated adverse cardiovascular events due to its influence on endothelial cell migration and endothelial wound repair23.

In conclusion, in the context of cardiac health this study showed negative as well as positive metabolic impacts associated with fermentation of the investigated seaweed extract concentrations, as compared with the negative control and the positive control inulin. Taken together with our SCFA- and metagenomic analysis, the results of this study suggest a net-positive health benefit of the seaweed extract in the gastro-intestinal tract.

In conclusion, the results of this study indicate that the gut microbiota of all five donors effectively fermented the **seaweed extract**. The extract stimulated the production of primary metabolite acetate, but not lactate. The product had propionogenic and butyrogenic effects, even at low concentration (0.5 g/L). Gas production was low and not likely to cause bloating, even at the highest concentration (3.5 g/L). **Inulin** strongly stimulated production of acetate, lactate, propionate, and butyrate. Gas production was stimulated to an extent potentially associated with discomfort in the host. For each of these markers, effects were more

pronounced as compared to seaweed extract, but in the case of butyrate the effect was subject to higher interindividual differences.

Metagenomics confirmed that the seaweed extract was well fermented by the gut microbiota of the five donors, as it induced an increase in bacterial biomass and species richness. Seaweed extract fermentation was associated with a compositional shift involving uncultured/unclassified *Oscillospiraceae*, *Butyricicoccus*, *Veillonellaceae* and/or *Lachnospiraceae* (related to *Eubacterium*). These enrichments explain the product's propionogenic and butyrogenic effects. Like the seaweed extract, inulin enriched propionate-producing *Veillonellaceae*, but also butyrate-producers, including *Blautia*, *Faecalibacterium* and unclassified *Lachnospiraceae*. The latter indicates that inulin's butyrogenic effect was attributed to the enrichment of bacteria other than those enriched by Oceanium's seaweed extract.

Metabolomic analysis showcased both negative as well as positive metabolic impacts associated with fermentation of the seaweed extract concentrations, with differential production of cardiac metabolites TMAO, TMA, carnitine, indole and *p*-cresol, among others. While metabolite levels were generally on par with levels observed in the negative control, seaweed extract treatment did generate beneficial metabolic shifts when compared to the fiber control inulin.
